# Interferons Inhibit Ebola Virus Infection of Human Keratinocytes

**DOI:** 10.3390/v17121577

**Published:** 2025-12-02

**Authors:** Jonah Elliff, Hanora Van Ert, Kristina Sevcik, Marija Anne Djurkovic, Danielle Rudd, Francoise Gourronc, Aloysius Klingelhutz, Olena Shtanko, Wendy Maury

**Affiliations:** 1 Department of Microbiology and Immunology, University of Iowa, Iowa City, IA 52242, USA; jonah-elliff@uiowa.edu (J.E.); hanora-vanert@uiowa.edu (H.V.E.); ksevcik@unmc.edu (K.S.); francoise-gourronc@uiowa.edu (F.G.); al-klingelhutz@uiowa.edu (A.K.); 2 Interdisciplinary Graduate Program in Immunology, University of Iowa, Iowa City, IA 52242, USA; 3 Host Pathogens Interactions, Texas Biomedical Research Institute, San Antonio, TX 78227, USA; djurkovic@livemail.uthscsa.edu (M.A.D.); oshtanko@txbiomed.org (O.S.); 4 Department of Biology, Mt. Mercy College, Cedar Rapids, IA 52402, USA; drudd@mtmercy.edu

**Keywords:** keratinocyte, Ebola virus, interferon-stimulated gene, antiviral, skin, type I interferon, type II interferon, type III interferon, filovirus

## Abstract

*Orthoebolavirus zairense* is the species name for Zaire Ebola virus (EBOV) within *Filoviridae*. This group of viruses can cause severe disease in humans, characterized by hemorrhagic shock, coagulation abnormalities, and severe inflammation. While tissue macrophages are critical targets early during EBOV infection, other cell types support viral replication as disease progresses. At late stages of infection, infectious EBOV is found on the surface of the skin, which may be a critical source of infectious virus transmitted between individuals during outbreaks. Human skin contains a number of cellular targets of EBOV, including keratinocytes. Here, we demonstrate EBOV infection of telomerase-immortalized normal human skin keratinocytes (NHSK-1), as well as EBOVΔVP30 infection of NHSK-1 cells that were stably complemented with EBOV transcription factor VP30. Infection with EBOVΔVP30 did not elicit detectable endogenous interferon responses; however, exogenous pre-treatment of NHSK-1 cells with type I, II, and III interferon (IFN) inhibited EBOVΔVP30 infection and infection of an additional low-containment model of EBOV, rVSV/EBOV GP, in a dose-dependent manner. Analysis of the transcriptome of IFN-treated keratinocytes identified multiple genes unique to each IFN and a subset of ISGs upregulated by all three IFNs. Our results indicate that ISGs induced by IFN pre-treatment of keratinocytes can reduce infection, underlining that ISGs may serve as EBOV-targeting therapeutics.

## 1. Introduction

Ebola virus (EBOV) is an enveloped, negative-sense RNA virus within the family *Filoviridae* that is responsible for devastating episodic outbreaks in equatorial Africa, with mortality rates ranging from 30–90% depending on the outbreak context [1,2,3]. The largest recorded outbreak occurred in West Africa in 2013–2016 and resulted in ~28,000 infections and ~11,000 deaths, inciting major global health concerns. Smaller episodic outbreaks have since occurred in the Democratic Republic of the Congo (DRC) and Guinea, with varied size and duration [3,4]. Infection with EBOV causes Ebola virus disease (EVD), an acute disease that is characterized by an aberrant and dysregulated immune response that destabilizes the vascular system. This results in a syndrome similar to fulminant septic shock, characterized by low blood pressure and widespread tissue ischemia, along with high fever; patients usually succumb due to multiorgan failure [5,6]. Some cutaneous manifestations have been described in EBOV-infected humans and non-human primates (NHPs), including a maculopapular rash and petechiae [7,8].

Non-therapeutic containment measures currently employed during outbreaks include disease surveillance, quarantine of infected individuals, and education on contact precautions [9]. Two vaccines, including an effective recombinant vesicular stomatitis virus-vectored vaccine that encodes the EBOV glycoprotein (rVSV/EBOV GP), are now licensed and recommended for use by the World Health Organization (WHO) [10,11,12]. Two monoclonal antibody regimens are also currently approved for the treatment of EVD: the antibody cocktail REGN-EB312 [13] and the single monoclonal antibody mAb11413 [14]. These treatments showed moderate efficacy in a large outbreak in the DRC [15] but can be prohibitively expensive in socio-disadvantaged areas where EVD outbreaks occur. Thus, efforts to identify additional therapies for treatment and control of viral transmission are ongoing.

Transmission of EBOV occurs through direct contact with an infected individual or their body fluids [16]. While mucosal transmission is considered to be the primary route [17], evidence suggests that direct skin-to-skin contact may also be an important route of transmission. During the 2013–2016 West Africa epidemic, studies demonstrated that infectious virus is present on the skin of infected individuals late during infection [17,18,19]. Detection of EBOV antigen and/or RNA in the skin and on the surface of skin at late stages of infection of humans and experimentally infected NHPs further suggest that infectious virus disseminates to the skin of infected individuals [7,17,18,19,20,21,22,23,24]. We previously explored EBOV-permissive cell types in the skin using ex vivo infections of intact human skin explants and in vivo infections of NHPs and mice [23,24]. In these studies, we defined keratinocytes as a skin cell that supports productive EBOV infection. Keratinocytes are the most abundant cell type found in the epidermis (the outermost layer of the skin) and are targets of multiple viruses, including human papillomavirus (HPV), beta- and gamma-herpesviruses, flaviviruses, and alphaviruses [25,26,27,28,29]. The role of keratinocytes in supporting replication of a broad array of viruses, the observation that EBOV directly infects human keratinocytes, and the detection of virus on the surface of skin implicates these keratinocytes in transmission of EBOV through direct skin contact. Therapeutics targeting replication of EBOV in keratinocytes may have utility in controlling contact transmission during outbreaks.

A number of studies suggest that interferons (IFNs) may provide a protective response against filovirus infection and transmission [30,31,32]. There are three main types of IFN-type I (IFN-α and -β), type II (IFN-γ), and type III (IFN-λ) - that elicit expression of large and partially overlapping sets of interferon-stimulated genes (ISGs) [33]. Type I interferons bind to the heterodimeric interferon-α/β receptor (IFNAR), inducing a signaling pathway that results in formation of a trimeric complex consisting of Signal Transducers and Activators of Transcription 1 and 2 (STAT1/2) and Interferon Regulator Factor 9 (IRF9). While this canonical pathway is well established, STAT-independent pathways can also be activated by type I IFNs and may elicit specific ISGs [34]. IFN-γ is the most structurally divergent from the other two classes of IFN and binds a different heterodimeric receptor comprised of interferon-gamma receptors 1 and 2 (IFNGR1/2) to elicit STAT1-dependent production of ISGs [35]. IFN-λ is the least studied member of the IFN family. It is primarily produced by epithelial cells and is structurally related to type I IFNs [35,36]. IFN-λ induces signaling through its own heterodimeric receptor consisting of interferon-lambda receptor 1 (IFNLR1) and IL10Rβ that elicits formation of the STAT1-STAT2-IRF9 complex to drive transcription of ISGs. In the context of viral infections, IFN signaling stimulates the production of hundreds of ISGs, many of which have direct antiviral activity against a broad array of viruses [37,38,39]. A subset of these ISGs can modulate the activity of immune cells, such as T cells, B cells, and/or dendritic cells (DCs) [40,41,42], bridging innate and adaptive immunity to enhance immune control of virus replication. Thus, topical application of IFNs might serve as an effective antiviral treatment against viruses that target the skin. However, it should be noted that IFN responses can be pathogenic and are implicated in multiple autoimmune disorders, including psoriasis and others that affect the skin [43,44,45,46,47].

Specific ISGs such as TRIM25, ISG15, and several IFITMs [48,49,50] have been shown to interfere with EBOV replication. A large screen of 400 ISGs showed that approximately 21 genes caused statistically significant decreases in replication of a low-containment EBOV model in HEK 293T cells [51], emphasizing the diversity of genes that can directly impact EBOV infection. The expression and function of certain ISGs can be cell-type dependent [52,53,54,55], underlining the importance of exploring ISGs and their associated antiviral functions in different permissive cell types. To date, the ability of IFN to inhibit EBOV in human keratinocytes has not been studied.

Here, we demonstrate that telomerase-immortalized keratinocytes from adult human skin support EBOV infection and infection with a low-containment model of EBOV, EBOVΔVP30. EBOVΔVP30 infection elicited no detectable endogenous interferon responses in human keratinocytes, in contrast to robust innate immune responses elicited by a different low-containment model of EBOV infection, rVSV/EBOV GP. We also show that IFN-β, IFN-γ, and IFN-λ elicit dose-dependent inhibition of EBOV replication in keratinocytes. Analysis of the transcriptome of IFN-treated keratinocytes identified a common subset of ISGs expressed in response to all three treatments, as well as ISGs uniquely stimulated by each specific IFN type. Further, we examined ISG production elicited by the IFNs at early (6 h) and late (24 h) time points. These findings identify an array of different ISGs that may serve as antivirals against EBOV in human keratinocytes and support the development of focused therapeutic strategies to reduce skin-transmissible EBOV infection during future outbreaks.

## 2. Materials and Methods

### 2.1. Cell Lines

The telomerase-immortalized normal human skin keratinocyte-1 (TERT-NHSK-1, herein referred to as NHSK-1) cell line was derived from normal keratinocytes of healthy human adult skin and has been previously described [56,57]. NHSK-1 cells stably expressing VP30 were generated by transduction of a VP30-encoding pBABE-puromycin retrovirus into NHSK-1 cells. Cells were selected with puromycin (1 μg/mL), and individual colonies were cloned via a ring-cloning approach. VP30 expression in clones was confirmed by an immunoblot using a rabbit α-EBOV VP30 polyclonal antiserum (0301-048, IBT Bioservices, Rockville, MD, USA). Clone C was selected based on growth kinetics and support of EBOV GP-dependent infection and will herein be referred to as NHSK-1-VP30 cells. Two other clonal populations (Clones A and B) are used in Figure S5B,C. Primary keratinocyte cultures were obtained from the skin of young, healthy adults using methods similar to those previously described [56]. All keratinocytes were maintained at 37 °C, 5% CO_2_ in keratinocyte serum-free media (K-SFM, Gibco, Waltham, MA, USA) supplemented with 25 µg/mL bovine pituitary extract, 0.16 ng/mL epidermal growth factor, and 1% penicillin/streptomycin. The keratinocyte medium was refreshed every other day, and the cells were utilized when they reached 70%–80% confluence.

VP30-expressing Vero cells have been previously described [58] and were used for generation of EBOVΔVP30 stocks. Vero E6 cells were utilized for generation of EBOV stocks. Both Vero E6 and Vero-VP30 cells were maintained at 37 °C, 5% CO_2_ in Dulbecco’s modified Eagle’s medium (DMEM, Gibco, Thermo Fisher Scientific, Waltham, USA) supplemented with 10% fetal bovine serum (FBS) and 1% penicillin/streptomycin.

### 2.2. Virus Stocks

EBOV: Experiments with replication-competent EBOV encoding green fluorescent protein (EBOV-GFP, herein referred to as EBOV) were performed in a maximum containment (BSL-4) laboratory at Texas Biomedical Research Institute (Texas Biomed, San Antonio, TX, USA) according to approved standard operating procedures and protocols approved by the institute’s Biohazard and Safety and Recombinant DNA Committees. The NCBI accession number for the EBOV used in these studies is KF990213. EBOV stocks were generated in Vero cells in DMEM supplemented with 2% FBS for 7 days. The culture supernatant was clarified of cell debris, then layered over a 20% sucrose cushion in PBS and centrifuged at 28,000 rpm at 4 °C for 2 h. The virus pellet was resuspended in PBS and stored at −80 °C until use. The virus was titrated by incubating Vero cells with serial dilutions of the stock for 24 h (h) and determining fluorescent foci units per ml (FFU/mL).

EBOVΔVP30: To amplify EBOVΔVP30 encoding GFP (EBOVΔVP30-GFP, herein referred to as EBOVΔVP30), Vero-VP30 cells were infected with a low MOI (~0.005). Infected cells were scraped and homogenized by pipetting up and down at 120 h following infection when robust GFP expression was observed. Supernatants containing virus and cell homogenates were then centrifuged at 1500× *g* for 5 min and passed through a 0.45 µm filter to remove cell debris. Filtered supernatants were either aliquoted and frozen at −80 °C or layered over a 25% sucrose/PBS cushion and ultracentrifuged at 28,000× *g* for 2 h for a sucrose-purified viral stock. Pellets were thoroughly resuspended in PBS on ice, aliquoted, and frozen at −80 °C until use. For some studies, filtered cell supernatants containing EBOVΔVP30 were used for infection, whereas other studies utilized sucrose-purified virus, as noted. Viral stocks were titrated by serial dilution and application to Vero-VP30 cells and evaluation of GFP-positive wells at 120 h following infection to obtain the TCID_50_.

rVSV/EBOV GP: To amplify rVSV/EBOV GP encoding GFP (rVSV/EBOV GP-GFP, herein referred to as rVSV/EBOV GP), Vero E6 cells were infected with a low MOI (~0.005). At 48 h post infection (hpi), the virus-containing supernatant was centrifuged at 1500× *g* for 5 min and passed through a 0.45 µm filter to remove cell debris. Filtered supernatants were layered over a 25% sucrose/PBS cushion and ultracentrifuged at 28,000× *g* for 2 h. Sucrose purified pellets were thoroughly resuspended in PBS, aliquoted, and frozen at −80 °C until use. Viral stocks were titered by serial dilution of the virus, application to Vero E6 cells, and evaluation of GFP-positive wells at 120 hpi to obtain the TCID_50_.

### 2.3. Infections

EBOV: NHSK-1 cells were seeded into 48-well plates at 10^5^ cells/well and were left uninfected or incubated with EBOV at a multiplicity of infection (MOI) of 0.1, 1, or 10 (as titrated in Vero cells). After 1 h, cells were washed twice with PBS, then incubated with fresh medium for 24, 48, 72, or 96 h. At each time point, the cells were fixed, and the supernatants were titrated in Vero cells. All cells were stained with Hoescht dye (Thermo Fisher) to stain nuclei and photographed using an automated Nikon Ti-Eclipse microscope (Nikon, Tokyo, Japan). The numbers of cell nuclei and infected (GFP-positive) cells were counted using CellProfiler v4.2.3 (Broad Institute). Infection efficiency in keratinocyte monolayers was quantified as the ratio of infected cells to cell nuclei. The virus titer in cell supernatants was determined in Vero cells and is represented as FFU/mL.

**Figure 1:**
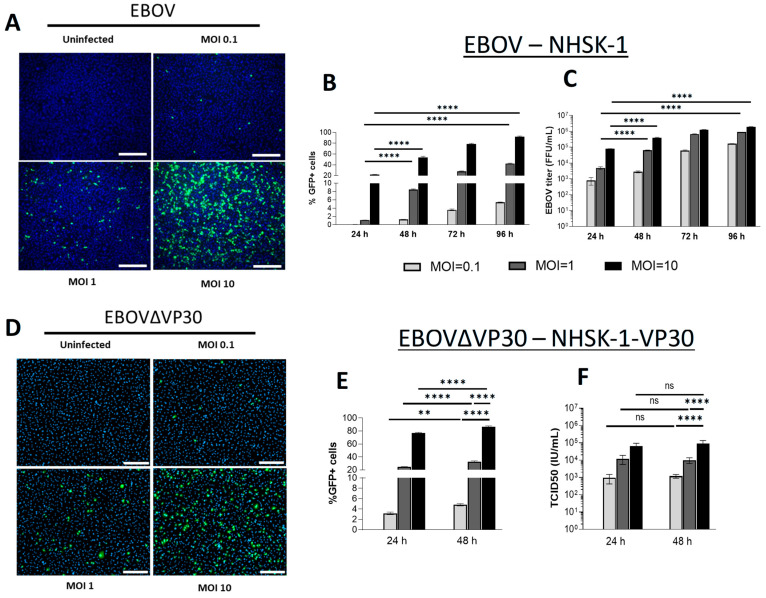
Immortalized keratinocytes support EBOV and EBOV∆VP30 infection. (**A**) Immortalized human keratinocytes (NHSK-1) were infected with increasing concentrations of EBOV (MOI = 0.1, 1, and 10), and inoculum was replaced with fresh media at 1 hpi. Representative images of infected cells were obtained at 48 hpi. Green = EBOV-GFP, blue = DAPI, scale bar = 300 μm. (**B**,**C**) Quantification of GFP+ EBOV-infected keratinocytes from image analysis (**B**) and supernatant EBOV titers (**C**) at 24, 48, 72, and 96 hpi (*n* = 3, data obtained from one experiment). (**D**) NHSK-1 cells were infected with increasing concentrations of EBOVΔVP30-GFP (MOI = 0.1, 1, and 10), and inoculum was replaced with fresh media at 4 hpi. Representative images of infected cells were obtained at 48 hpi. Green = EBOVΔVP30-GFP, blue = DAPI, scale bar = 200 μm. (**E**,**F**) Quantification of GFP+ EBOVΔVP30-infected keratinocytes by flow cytometry (**E**) and supernatant EBOVΔVP30 titers (**F**) are shown at 24 and 48 hpi (*n* = 4, data are representative of two independent experiments). Data in (**B**,**C**,**E**,**F**) were analyzed using two-way ANOVA with Tukey’s multiple comparisons test. Data shown on a log_10_ scale were log_10_-transformed prior to analysis. Data are presented as mean ± SD. ** *p* < 0.01, **** *p* < 0.0001, ns = not significant.

EBOVΔVP30 and rVSV/EBOV GP: For the EBOVΔVP30 studies, NHSK-1-VP30 cells were seeded in 48-well plates at a density of 10^5^ cells/well and left uninfected or incubated with EBOVΔVP30 at an MOI of 0.1, 1, or 10 for 24 or 48 h at 37 °C, 5% CO_2_. For most studies, inoculum was added to cells and not removed for the duration of infection. In the studies shown in Figure 1, the EBOVΔVP30 inoculum was replaced with fresh media at 4 hpi, and the media containing the virus was collected at the indicated time points and titrated in Vero-VP30 cells to assess production of new infectious virus. In the rVSV/EBOV GP studies, NHSK-1 cells or primary keratinocytes were plated as described above and incubated with rVSV/EBOV GP at an MOI of 10 for 24 h.

For assessing percent infection, cells were washed with sterile PBS and detached with 0.05% Trypsin-EDTA (37 °C for 15 min). PBS supplemented with 2% FBS was added to inactivate Trypsin-EDTA. For the EBOVΔVP30 studies, the cells were centrifuged at 700× *g* for 5 min, resuspended in 4% paraformaldehyde (PFA), and incubated at 4 °C for 30 min to inactivate residual EBOVΔVP30. The cells were then washed in FACS buffer (2% FBS/PBS) and resuspended in FACS buffer. The frequency of GFP+ cells was assessed via flow cytometry on a BD FACSVerse or Beckman CytoFlex instrument, and the data were analyzed using FlowJo v10.8.0 (BD Biosciences, Franklin Lakes, NJ, USA). The gating strategy is shown in Figure S1. For representative images, cells were fixed on coverslips with 4% PFA, then stained with anti-GFP-AF488 (Invitrogen) and DAPI for 1 h at room temperature. The cells were washed, mounted on slides, and imaged with an Axio Observer 7 microscope (Carl Zeiss AG, Oberkochen, Germany).

### 2.4. Treatment with IFN

Human IFN-α (PBL Assay Science, Piscataway, NJ, USA), human IFN-β (R&D Systems, Minneapolis, MN, USA), human IFN-γ (PBL Assay Science), and human IFN-λ (PBL Assay Science) were used in these studies. Semi-confluent (70%–80%) cultures of NHSK-1-VP30 cells were treated with the concentration and type of IFN noted in each figure legend or K-SFM alone (control) for 6 or 24 h and washed gently with sterile PBS, and the medium was replaced. Cultures were either infected with EBOVΔVP30 or rVSV/EBOV GP as described above, or RNA was isolated for transcriptomic studies. For the viability studies, cells that were treated with IFN for 24 h were detached as described above for infections. The cells were centrifuged at 700× *g* for 5 min, then resuspended in viability dye (1:1000 dilution in PBS; Fixable Viability Dye eFluor450; eBioscience) for 15 min at room temperature. The cells were then washed in FACS buffer and resuspended in FACS buffer. The frequency of live cells was assessed via flow cytometry on a BD FACSVerse or Beckman CytoFlex instrument, and the data were analyzed using FlowJo software.

### 2.5. Lentiviral ISG Overexpression

Lentivirus expression clones containing cDNA sequences for human IRF1, GBP5, ISG15, CMPK2, or TRIM22 were co-transfected into HEK293T cells using the JetPRIME transfection protocol (Sartorious, Göttingen, Germany) with a second-generation lentiviral packaging plasmid, psPAX2 (Addgene, Watertown, MA, USA) and a plasmid encoding the vesicular stomatitis virus glycoprotein (VSV-G). At 24, 48, and 72 h post-transfection, the filtered supernatant was layered over a 25% sucrose/PBS cushion and ultracentrifuged at 28,000× *g* for 2 h for a sucrose-purified lentivirus stock.

NHSK-1-VP30 cells were seeded in 48-well plates at a density of 10^5^ cells/well. When confluent, cells were incubated with equivalent volumes of the indicated purified lentivirus for 6 h in the presence of a JAK/STAT inhibitor (Ruxolitinib). The inoculum was replaced with fresh medium, and the cells were infected 48 h later with MOI = 10 EBOVΔVP30. At 48 hpi, infection was evaluated as described above.

### 2.6. Endogenous Antiviral Response to Infection

NHSK-1-VP30 cells were seeded at a density of 7 × 10^5^ cells/well in a six-well format. When 90% confluent, the medium was replaced with either medium alone (uninfected) or medium containing purified EBOVΔVP30 at an MOI = 10 or rVSV/EBOV GP at an MOI = 10. At 24 hpi, the medium was removed, and cellular RNA was isolated using a Qiagen RNeasy Mini Kit as described by the manufacturer. In parallel wells, infection was assessed via flow cytometry, as described above. Antiviral genes were analyzed using real-time PCR with Human Antiviral Response RT2Profiler™ PCR array plates from Qiagen (PAHS-122ZC-24). RT-qPCR was performed using a QuantStudio3 cycler (Applied Biosystems, Waltham, MA, USA), with the cycling conditions described by the manufacturer. Analysis was done using the data analysis spreadsheet provided by the manufacturer.

### 2.7. Transcriptomic Analysis of IFN-Treated NHSK-1 Cells

Two individual studies were performed using parental NHSK-1 cells. The cells were plated to semi-confluency (70–80%) in a six-well format and subsequently received one of the following treatments. In one study, 100 ng/mL of IFN-β, 100 ng/mL of IFN-γ, or medium only was added to wells. In a second study, 2.47 ng/mL IFN-α, 18.8 ng/mL IFN-λ, or medium only was added. All treatments were carried out for 6 and 24 h. All treatments were performed in quadruplicate at each time point. RNA was extracted from cells utilizing the RNeasy^®^ Mini Kit from Qiagen as described by the manufacturer. High-quality RNA samples were verified by a Bioanalyzer (Agilent) and used as input to generate mRNA-seq libraries for the Illumina platform at the Iowa Institute of Human Genetics (IIHG), Genomic Division. The libraries were sequenced on a Novaseq 6000 (Illumina) system utilizing two lanes on an SP flow cell. Paired-end reads were utilized at a read depth of ~400 M. All downstream processing of RNA-seq reads and bioinformatic analysis were conducted utilizing RStudio (v. R 4.1.1), accessed through the University of Iowa Interactive Data Analysis Service utilizing the high-performance computing cluster as needed. Adapter sequences from the raw FASTQ files were trimmed utilizing Trim Galore! (v.0.0.6; Babraham Bioinformatics—Trim Galore!) and analyzed with the included FastQC program. All samples met the quality control measure of a Phred score of ≥20. Utilizing Salmon [59] (v.1.5.2), we created a decoy-aware transcriptome utilizing the whole human genome as a decoy (Selective Alignment (combine-lab.github.io)), using the *Homo Sapiens* genome and annotated files from Gencode (GRCh38.p13; Gencode.v.38.annotatio.gtf). Gene counts were obtained utilizing aligned reads and the Salmon quant feature with the—validateMappings, —seqBias, and—gcBias commands. All samples resulted in ≥80% of reads mapping to our generated index and were utilized in differential gene expression (DEG) analysis. DEG analyses were conducted utilizing DESeq2 (v.1.34.0) with the design formula “~interferon + time + interferon:time” [60]. In each separate experiment, we compared individual interferon treatments to the untreated control that was initially included with each RNA isolation (none compared with IFN-β and IFN-γ and none1 compared with IFN-α and IFN-λ). Unless otherwise noted, we selected genes with Log_2_Fold Change values ≤−2 or ≥2 and a p-adjusted value of ≤10^−32^. Gene Ontology plots were created utilizing ClusterProfiler (v.4.2.2) [61,62]. Volcano plots were created utilizing Enhanced Volcano (v.1.12.0; GitHub—kevinblighe/EnhancedVolcano).

### 2.8. RNA Isolation and RT-qPCR

For RNA isolation, cells were harvested in TRIzol reagent (Invitrogen), followed by RNA extraction according to the manufacturer’s specifications. Next, 1 μg of RNA from each sample was converted to cDNA using a High-Capacity cDNA RevTrans Kit (Applied Biosystems). Quantitative PCR was performed using POWER SYBR Green Master Mix (Applied Biosystems) according to the manufacturer’s specifications. Data were collected on a QuantStudio 3 Real Time PCR instrument, and Ct values were determined with QuantStudio Data Analysis software (Applied Biosystems). Averages from duplicate wells for each gene were used to calculate the abundance of transcripts relative to the housekeeping gene Gapdh and are presented as 2-ΔCt. Primers were obtained from Integrated DNA Technologies, Coralville IA, and the sequences are as follows: SLC15A3 fwd 5′-TGGCGTTTATTCAGCAGAACA-3′, SLC15A3 rev 5′-TCTCTGGCCGAGTGTCGTT-3′, CMPK2 fwd 5′-CCAGGTTGTTGCCATCGAAG-3′, CMPK2 rev 5′-CAAGAGGGTGGTGACTTTAAGAG-3′, BATF2 fwd 5′-AGACCCCAAGGAGCAACA-3′, BATF2 rev 5′-CAGGGCGAGGTTGTCTTT-3′, GAPDH fwd 5′-TTAAAAGCAGCCCTGGTGAC-3′, GAPDH rev 5′-CTCTGCTCCTCCTGTTCGAC-3′.

### 2.9. Quantification and Statistical Analysis

All statistical analysis was performed using GraphPad Prism v9.4.1 (GraphPad, San Diego CA). Statistical analysis for the RNA sequencing datasets is described above. Quantification of flow cytometry data was performed using FlowJo v10.8.0 (Becton, Dickinson & Company, Ashland OR). Where indicated, statistical significance is defined as * *p* < 0.05, ** *p* < 0.01, *** *p* < 0.001, **** *p* < 0.0001, ns = not significant. Specific details regarding the statistical tests used and *n* values for each experiment can be found in the corresponding figure legends.

## 3. Results

### 3.1. Human Keratinocytes Support EBOV and EBOV∆VP30 Infection

We have previously shown that primary human keratinocytes are permissive to EBOV infection [23]. A telomerase-immortalized human keratinocyte cell line derived from adult human skin (normal human skin keratinocyte-1 (NHSK-1)) was also previously shown to support EBOV GP-mediated viral infection of keratinocytes [23]. To demonstrate that this cell line is permissive to authentic EBOV replication, NHSK-1 cells were infected with increasing doses of EBOV encoding GFP [63]. NHSK-1 cells supported dose-dependent infection with EBOV (Figure 1A) and spread of infection throughout the monolayer was observed from 24 to 96 hpi (Figure 1B). Production of infectious EBOV was also evident in supernatants of infected cells at each time point (Figure 1C), further demonstrating that NHSK-1 cells are permissive for productive EBOV infection.

We evaluated whether NHSK-1 cells could support infection of an established low-containment model virus of EBOV that lacks the viral transcriptional activator, VP30 (EBOVΔVP30) [58]. Deletion of VP30 from the viral genome restricts viral replication to VP30-expressing cell lines, resulting in a biologically contained virus that is a low-risk approach to studying viral replication and cell-intrinsic immune responses to infection. A GFP reporter is expressed in the place of VP30, allowing easy readout of viral replication. NHSK-1 cells that stably expressed VP30 (NHSK-1-VP30) supported dose-dependent infection with EBOVΔVP30 (Figure 1D) and resulted in both viral antigen and infectious virus in the supernatant (Figure 1E,F). While spread of EBOVΔVP30 infection within the monolayer was observed between 24 and 48 hpi (Figure 1E), these increases were modest. Notably, infection as detected by GFP was greater in EBOVΔVP30-infected cells at 24 hpi (Figure 1E) than that observed in EBOV-infected cells (Figure 1B) at all MOIs used. Given the high percentage of infected cells and substantial viral gene expression as indicated by GFP, EBOVΔVP30 is a useful low-containment approach for studying various aspects of the EBOV lifecycle, as well as cellular innate immune responses to EBOV in keratinocytes. Together, these studies demonstrate that human keratinocytes support productive replication of EBOV and EBOVΔVP30.

### 3.2. EBOVΔVP30 Infection Does Not Stimulate Innate Antiviral Responses in Human Keratinocytes

**Figure 2:**
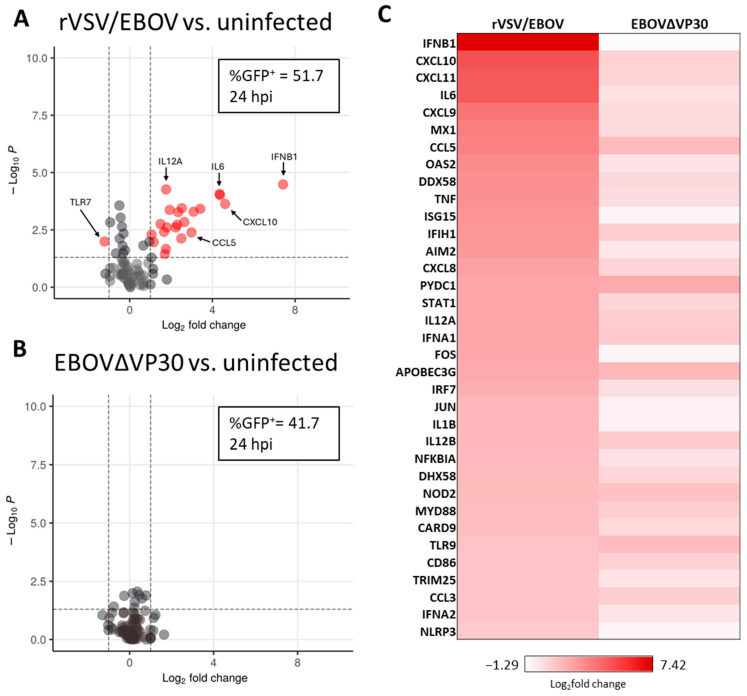
EBOVΔVP30 infection does not stimulate innate antiviral responses in human keratinocytes. NHSK-1 cells expressing EBOV VP30 were infected with rVSV/EBOV or EBOV∆VP30 (MOI = 10) or left uninfected. The percent of virus-infected cells was evaluated at 24 hpi by flow cytometry of GFP^+^ cells. RNA was isolated from the cultures for assessment of antiviral gene expression via PCR at 24 hpi. (*n* = 3, representative of two independent experiments). (**A**,**B**) Volcano plot showing upregulation of genes in (**A**) rVSV/EBOV-infected cells or (**B**) EBOV∆VP30-infected cells compared with uninfected cells. The percentage of GFP^+^ cells was determined in parallel by flow cytometry. The significance cut-off is defined as *p* < 0.05 (vertical dotted lines), and the cut-off for differential expression is defined as a log_2_fold > 1 (horizontal dotted lines). Genes represented in red meet the significance and differential expression cutoffs. Significance was calculated using Student’s *t*-test. (**C**) Heatmap showing the top 35 innate immune-associated genes upregulated at 24 h with rVSV/EBOV compared with expression at 24 h with EBOV∆VP30 infection.

EBOV encodes two proteins with IFN antagonistic activity, VP24 and VP35, to circumvent innate immune recognition and induction of endogenous antiviral responses [64,65,66,67]. Although these proteins effectively block IFN responses, it is plausible that the IFN pathway in some cell types may not be effectively inhibited, given that in vivo infections can elicit early innate immune responses [68,69]. We leveraged the EBOVΔVP30 model to determine whether EBOV infection elicits cell-intrinsic antiviral responses in human keratinocytes. NHSK-1 cells were infected with EBOVΔVP30 or another low-containment virus model, vesicular stomatitis virus encoding EBOV GP and GFP in the place of native G glycoprotein (rVSV/EBOV GP). We previously demonstrated that rVSV/EBOV GP productively infects NHSK-1 cells, as well as primary human keratinocytes and keratinocytes, within the epidermis of human skin explants [23]. As rVSV/EBOV GP elicits protective innate immunity against EBOV [70,71], we anticipated that it would elicit strong IFN responses. At 24 hpi, when approximately 40–50% of cells were infected (Figure 2A,B), RNA was extracted, and 84 genes associated with antiviral responses were evaluated via RT-qPCR. Visualization of these data via volcano plot analysis showed that rVSV/EBOV GP infection elicited robust expression of IFN-β and multiple well-established ISGs such as CXCL10, MX1, and OAS2 (Figure 2A and Figure S2), as well as a significant decrease in expression of TLR7 RNA. In contrast, EBOVΔVP30 infection elicited no significant alteration in expression of these genes (Figure 2B and Figure S3). Comparison of the most highly upregulated genes in rVSV/EBOV GP-infected cells versus EBOVΔVP30-infected cells further demonstrated that there is little antiviral response in NHSK-1 cells to EBOVΔVP30 by 24 hpi (Figure 2C). Some genes were significantly different at 24 hpi with EBOVΔVP30 infection (ATG, CTSS, IFNAR1; Figure S3); however, the differences in expression were small. Although not significant, we observed larger trending increases in several genes such as CCL5, PYDC1, and APOBEC3G in EBOVΔVP30-infected cells (Figure S3). In separate studies, we found that expression of IFN-β was upregulated at 24 and 48 hpi during rVSV/EBOV GP infection but did not rise above baseline levels during EBOVΔVP30 infection from 6 hpi through 48 hpi, further supporting the absence of antiviral responses to EBOVΔVP30 (Figure S4). These studies demonstrate that EBOVΔVP30 infection of and replication in NHSK-1-VP30 cells is immunologically quiescent, indicating that EBOV is effective at blocking the initiation of innate immune responses in keratinocytes.

### 3.3. Exogenous Treatment with IFN-β, IFN-γ, and IFN-λ Inhibits EBOV Replication in Human Keratinocytes

**Figure 3:**
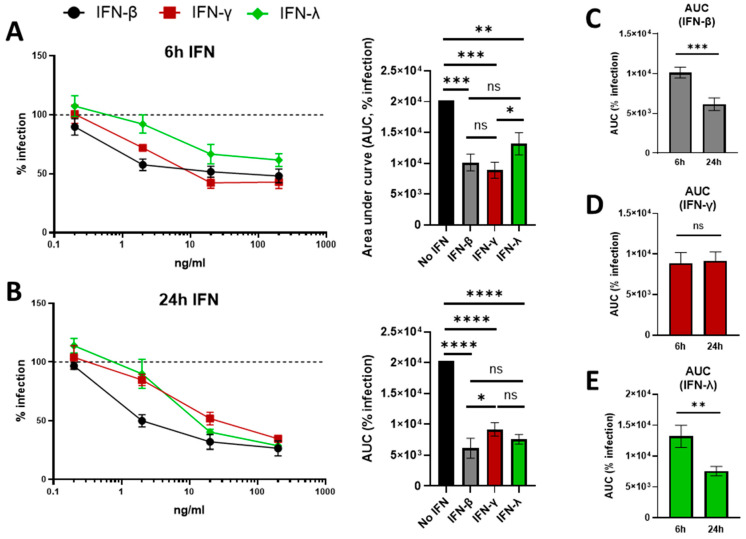
Treatment with IFN-β, IFN-γ, or IFN-λ inhibits EBOV∆VP30 replication in human keratinocytes. NHSK-1 cells expressing EBOV VP30 were treated with the indicated concentrations of IFN-β (black circles), IFN-γ (red squares), or IFN-λ (green diamonds) for 6 h or 24 h, then infected with MOI = 10 EBOV∆VP30 for 48 h. (**A**) EBOV∆VP30 infection of cells pre-treated for 6 h with IFN-β, IFN-γ, or IFN-λ evaluated by flow cytometry and compared to infection of non-treated cells (dotted line). Area under the curve (AUC) is shown (right). (*n* = 4, data pooled from two independent experiments). (**B**) EBOV∆VP30 infection of cells pre-treated for 24 h (open squares) with IFN-γ evaluated by flow cytometry and compared to infection of non-treated cells (dotted line). AUC is shown (right). (*n* = 4, data pooled from two independent experiments). (**C**,**D**) AUC values from (**A**,**B**), comparing infection of keratinocytes treated with IFN-β (**C**), IFN-γ (**D**), or IFN-λ (**E**) for 6 h or 24 h. Percent infections in (**A**,**B**) were analyzed by two-way ANOVA with Tukey’s multiple comparisons test and are presented as mean ± SD. AUC values were analyzed by one-way ANOVA (**A**,**B**) or Student’s *t*-test (**C**–**E**) and are presented as mean ± SEM. * *p* < 0.05, ** *p* < 0.01, *** *p* < 0.001, **** *p* < 0.0001, ns = not significant.

Both type I and type II IFNs have been demonstrated to inhibit EBOV replication in a variety of cell types, including macrophages, Huh7 cells, 769-P cells, and Vero cells [31,72,73]. However, the ability of IFNs to inhibit EBOV infection of human keratinocytes has not been examined. Therefore, we evaluated the effect of recombinant IFN treatments on EBOVΔVP30 infection of NHSK-1-VP30 cells. We included IFN-λ, as it is produced on epithelial surfaces [36], and its activity has been poorly evaluated in the context of EBOV infection. Cells were pre-treated with increasing concentrations of IFN-β, IFN-γ, or IFN-λ for 6 h or 24 h. Critically, we found that treatment for 24 h with 200 ng/mL (the highest dose used) of each IFN had no impact on cell viability (Figure S5A). After treatment, the cytokine-containing medium was replaced, and the cultures were infected with EBOVΔVP30 (MOI = 10). Treatment for 6 h with IFN-β, IFN-γ, or IFN-λ caused significant dose-dependent decreases in EBOVΔVP30 infection (Figure 3A). Both IFN-β and IFN-γ were similarly inhibitory at 6 h, while IFN-λ was significantly less inhibitory. Dose-dependent decreases in EBOVΔVP30 infection were also observed for all three IFNs when treatment was extended to 24 h (Figure 3B), with IFN-β and IFN-λ both conferring similar levels of inhibition. Notably, IFN-β was significantly more inhibitory than IFN-γ during a 24 h treatment. Total inhibitory activity of the IFNs against EBOVΔVP30 infection at 6 h and 24 h was assessed by determining areas under the curves (AUCs) (Figure 3C–E). Treatment for 24 h significantly enhanced the inhibition conferred by IFN-β and IFN-λ (Figure 3C,E), indicating an increase in EBOV-inhibitory ISG expression between 6 h and 24 h. The inhibition conferred by IFN-γ was similar between 6 h and 24 h (Figure 3D), providing evidence that expression of IFN-γ-elicited, EBOV-inhibitory ISGs had already occurred by 6 h. Inhibition by each IFN at 24 h of treatment was validated in other NHSK-1-VP30 clonal populations (Figure S5B,C).

Pre-treatment for 24 h with each IFN caused a dose-dependent inhibition of rVSV/EBOV GP as well, in both the immortalized NHSK-1 cell line and primary human keratinocytes (Figure S5D,E). At this time point, the ability of IFN-γ to inhibit rVSV/EBOV GP compared to type I IFN was reduced in these cells (Figure S5D), highlighting that immortalized keratinocytes may be weakly responsive to IFN-γ in the context of EBOV GP-mediated infection. However, rVSV/EBOV GP was strongly inhibited by IFN-β and IFN-γ in primary keratinocytes (Figure S5E), suggesting that this donor may be particularly sensitive to IFN treatment, or that immortalization dampens IFN responses that inhibit rVSV/EBOV GP replication.

Together, these data highlight the ability of type I, II, and III IFNs to inhibit EBOVΔVP30 infection of NHSK-1-VP30 cells and demonstrate a differential impact of treatment duration on EBOVΔVP30 inhibition with either type I/III or type II IFNs.

### 3.4. Interferons Elicit a Range of Overlapping and Unique ISGs in Human Keratinocytes

**Figure 4:**
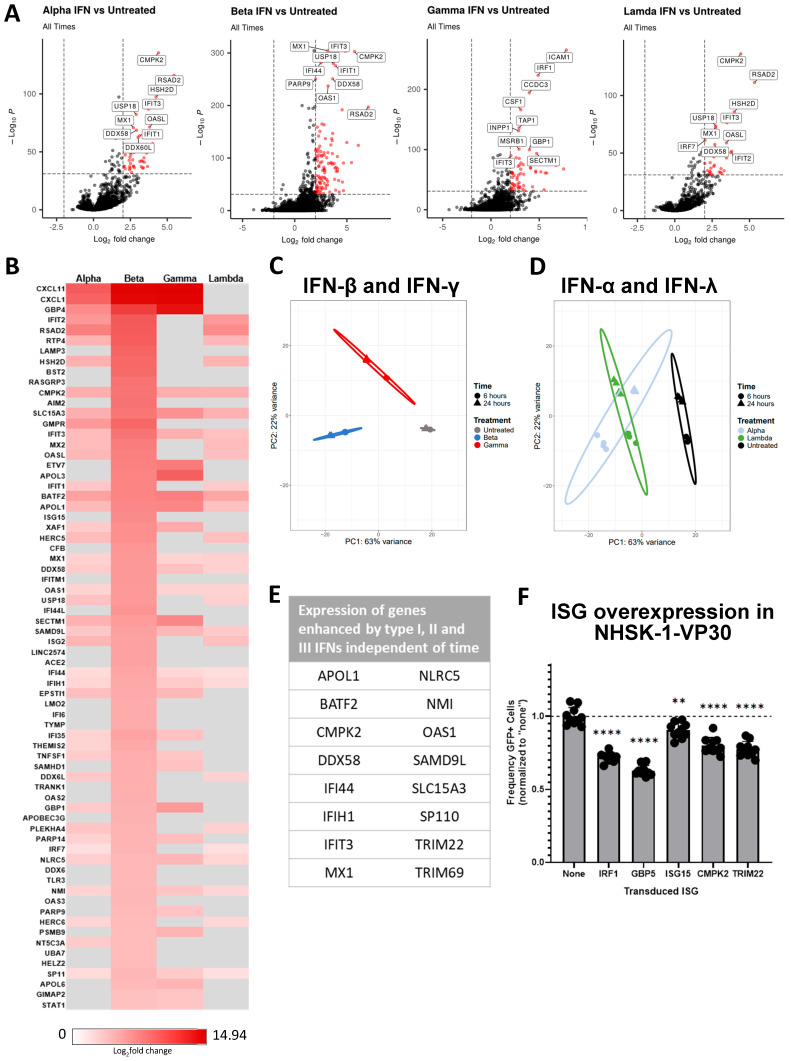
Interferons elicit a range of overlapping and unique ISGs in human keratinocytes. NHSK-1 cells were treated with 100 ng/mL of IFN-β or IFN-γ, 18.8 ng/mL of IFN-λ, or 2.47 ng/mL of IFN-α. RNA was isolated after 6 h or 24 h of IFN treatment. Sequencing was performed twice with four technical replicates for each IFN treatment. (**A**) Volcano plot analysis showing the highest upregulated genes represented in each IFN treatment, regardless of time. Genes highlighted in red are those that were identified based on a log_2_fold change of 2 or greater (vertical dashed lines) with a *p*-value of 10^−32^ (horizontal dashed lines). (**B**) Heatmap analysis of highest upregulated genes in IFN-β-treated cells compared to IFN-γ- and IFN-λ-treated cells. Grey boxes indicate that the gene did not meet the stringent criteria with the indicated IFN treatment. (**C**) PCA analysis of IFN-β- and IFN-γ- treated cells for each IFN at 6 h or 24 h. (**D**) PCA analysis of IFN-α- and IFN-λ- treated cells for each IFN at 6 h or 24 h. (**E**) Sixteen ISGs highly upregulated by all four IFNs at a log_2_fold change ≥2 and a *p*-value ≤10^−32^. (**F**) NHSK-1-VP30 cells were transduced with lentiviral constructs expressing the indicated genes, then infected with MOI = 10 EBOVΔVP30. Infection was evaluated at 48 hpi by flow cytometry. (*n* = 9–10, data pooled from two independent experiments.) Percent infections relative to untreated cells were analyzed using one-way ANOVA and are presented as mean ± SD. ** *p* < 0.01, **** *p* < 0.0001, ns = not significant.

With the appreciation that all three types of IFNs significantly reduced EBOVΔVP30 infection, we sought to investigate the breadth of IFN responses in human keratinocytes via bulk transcriptomic sequencing (RNA-seq). NHSK-1 cells were treated for 6 h or 24 h in these studies. In the first set of studies, cells were treated with 100 ng/mL of IFN-β or IFN-γ, and, in a separate experiment, cells were treated with IFN-λ (18.8 ng/mL) or IFN-α (2.47 ng/mL). These concentrations of IFNs are equivalent based on specific activity units/mL (U/mL) and have been converted to ng/mL concentrations for this study. The transcriptome of untreated cells was used as the baseline for gene expression, allowing identification of differentially expressed genes (DEGs) elicited by each IFN. Expression changes were initially compared independent of treatment duration, and DEGs were identified using highly stringent criteria: only genes with a log_2_fold change ≥2 and a *p*-value ≤ 10^−32^ were selected. This set of strict criteria limited the number of DEGs to a more manageable size. The pooled 6 h and 24 h transcriptomes of cells treated with each of the IFNs were distinct from the baseline untreated cells (Figure 4A). Many of the same genes were upregulated by IFN-α, IFN-β, and IFN-λ, including upregulation of ISGs typically associated with type I and III IFN treatment such as CMPK2, IFIT1, and RSAD2. In the IFN-γ treated cells, several well-known type II IFN-elicited genes such as IRF1 and TAP1 were also observed (Figure 4A).

In a heatmap analysis, we aligned the top 100 genes upregulated by IFN-β and compared these findings to the expression changes induced by other IFN treatments (Figure 4B). Several of the most robust hits for IFN-β were also upregulated by IFN-α and IFN-λ, whereas the pattern of altered expression differed in the IFN-γ-treated cells. To validate that the NHSK-1 cells used in the RNA-seq studies and the NHSK-1-VP30 cells used in our EBOVΔVP30 studies responded to IFNs in a similar manner, we assessed CMPK2 expression as a representative ISG. We observed that similar CMPK2 expression was induced in both NHSK-1 and NHSK-1-VP30 cells treated with IFN-β, IFN-γ, or IFN-λ (Figure S6A–C). These findings validate that the clonal VP30-expressing population retains the ability to respond to IFN.

By principal component analysis (PCA) performed for each independent experiment (Figure 4C,D), we observed that IFN-treated cells clustered distinctly from the associated untreated cells. Further, the 6 h and 24 h samples for each IFN treatment were distinct, but were well-separated from the untreated samples. Separation of the IFN-β and IFN-γ clusters was notable, particularly in the second dimension that represented 21% of the variance (Figure 4C). This is consistent with known differences between type I and type II IFN ISGs [35]. IFN-α and IFN-λ, however, significantly overlapped in the PCA, which was expected, given the similarities between type I and type III IFN signaling [36]. These data also demonstrate that the transcriptome differed more between types of IFN, as opposed to the differences caused by treatment duration.

In this analysis, 16 DEGs were found to be significantly upregulated by all interferon treatments (Figure 4E). These include genes encoding RNA sensors (DDX58, IFIH1) and well-established antiviral effector proteins (MX1, IFIT3, OAS1, and TRIM22). Several of these shared genes, such as SLC15A3, CMPK2, and BATF2, have not been explored in the context of EBOV infection. These genes were confirmed to be highly upregulated by 24 h treatment with a range of different IFN-β and IFN-γ concentrations (Figure S6D–F).

To test the functional relevance of these ISGs in the context of EBOV infection, we utilized a lentivirus overexpression system in NHSK-1-VP30 cells. We included several ISGs that are known to inhibit EBOV in other cell types (IRF1, GBP5, and ISG15) [31,49], as well as two candidate ISGs identified in our analysis (CMPK2 and TRIM22). Following transduction, the cells were infected with EBOVΔVP30. We found that both IRF1 and GBP5 reduced infection in NHSK-1-VP30s (Figure 4F), demonstrating that the EBOV-inhibitory capabilities of these ISGs extends to human keratinocytes. ISG15 had a more modest impact on infection, which is consistent from the complex role of the protein. CMPK2 and TRIM22 also modestly reduced infection, indicating that these proteins may have anti-EBOV capabilities and should be further evaluated in future studies.

In each IFN treatment, a biological pathways analysis revealed “response to virus”, “defense response to virus”, and “defense response to symbiont” as the top upregulated biological processes (Figure S7A–D). While all IFNs stimulated overlapping pathways involved in defense responses and regulation of viral replication, IFN-γ stimulated a lower percentage of genes involved in the top three antiviral pathways than the three other IFN types, as indicated by a lower GeneRatio value. IFN-γ also elicited pathways that were unique among the four IFN types tested, including “antigen processing and presentation”, which is consistent with the well-defined role of IFN-γ in orchestration of adaptive immune responses [42,74]. Molecular function analysis demonstrated that IFN-α, IFN-β, and IFN-λ treatment upregulated genes involved in double-stranded RNA binding and RNA helicase activity (Figure S7E–G). In contrast, IFN-γ stimulated unique pathways, including nucleoside binding pathways and regulation of signaling receptors (Figure S7H).

In total, these analyses confirm that NHSK-1 cells exhibit robust and somewhat overlapping ISG responses to IFN-α, IFN-β, IFN-γ, and IFN-λ and identify several ISGs with potential EBOV-inhibitory capabilities.

### 3.5. IFN Elicits Differential Gene Expression Patterns Between 6 and 24 h of Treatment

**Figure 5:**
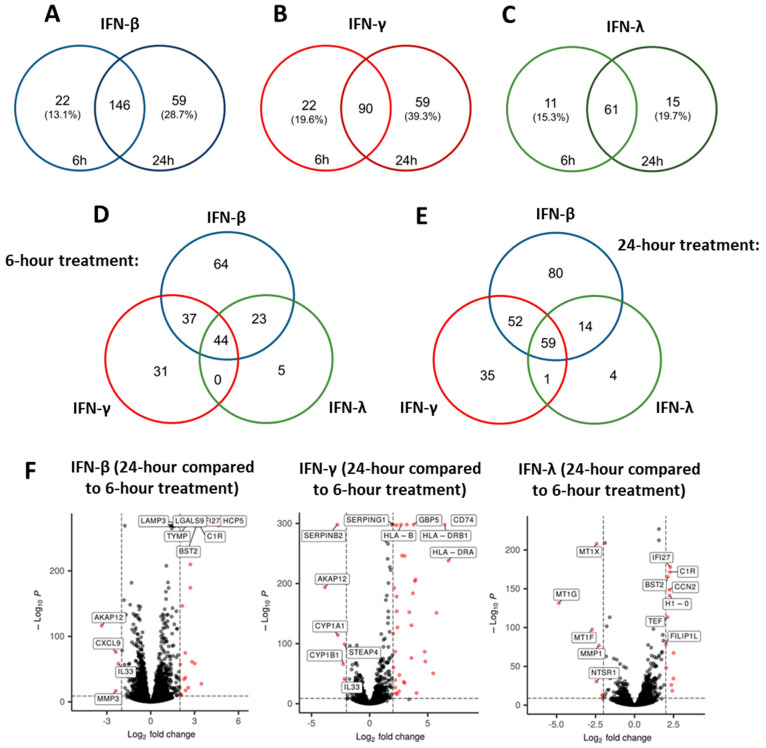
Timing of ISG upregulation following IFN treatment. NHSK-1 cells were treated with 100 ng/mL of IFN-β or IFN-γ or 18.8 ng/mL of IFN-λ. RNA was isolated for sequencing after 6 h or 24 h of IFN treatment. (**A**–**C**) Venn diagram comparing the number of shared and unique ISGs at 6 h or 24 h of treatment with IFN-β (**A**), IFN-γ (**B**), or IFN-λ (**C**). Percentages showing the fraction of unique DEGs at each time point out of the total DEGs at each time point. (**D**) Venn diagram depicting the number of shared and unique ISGs between each IFN treatment at 6 h only. (**E**) Venn diagram depicting the number of shared and unique ISGs between each IFN treatment at 24 h only. (**F**) Volcano plots comparing top upregulated genes at 24 h of treatment relative to 6 h treatment with each individual IFN type (IFN-β, left; IFN-γ, middle; IFN-λ, right). Vertical dashed lines refer to log_2_fold change of 2, and horizontal dashed lines refer to p-adjusted value of 0.05. Red dots indicate genes that meet these criteria.

Given that IFN-β- and IFN-λ-mediated control of EBOVΔVP30 replication was dependent on treatment duration (Figure 3), we examined the differential expression of genes between 6 h and 24 h of IFN treatment with type I, II and III IFNs. Based on the observation that IFN-α-stimulated ISGs comprised a subset of those stimulated by IFN-β treatment, IFN-β was leveraged as the representative type I IFN. We again used stringent criteria (log_2_fold change of 2 or greater and a *p*-value ≤10^−32^) to identify the most strongly upregulated DEGs at each treatment duration. In IFN-β-treated keratinocytes, we found 146 DEGs upregulated at both 6 h and 24 h of IFN treatment (Figure 5A and Table S1). We also identified 22 unique DEGs expressed at 6 h and 59 unique DEGs expressed following 24 h of treatment. Both IFN-γ and IFN-λ showed a substantial percentage of shared DEGs expressed at both 6 h and 24 h, with a smaller subset of DEGs that were unique to either 6 h or 24 h IFN treatment (Figure 5B,C, Tables S2 and S3).

Given that 24 h treatment with IFN-β and IFN-λ enhanced inhibition of EBOVΔVP30 infection over 6 h treatment (Figure 3), DEGs uniquely expressed between 6 h and 24 h of treatment may directly or indirectly inhibit EBOV infection. When comparing each IFN treatment, we found that 44 DEGs were shared by all three IFNs at 6 h of treatment (Figure 5D), and 59 DEGs were shared by all three IFN at 24 h of treatment (Figure 5E).

Using volcano plot analysis, we compared DEGs expressed at 24 h to those expressed at 6 h of IFN treatment (Figure 5F), finding unique sets of DEGs in each IFN treatment that were only expressed after 24 h of treatment. Three genes with the greatest differential upregulation following 24 h of IFN-β and IFN-λ treatment were the common ISGs IFI27, C1R, and BST2, whereas the most significantly upregulated genes by IFN-γ were distinct and were primarily genes involved in stimulating antigen presentation (CD74, HLA-B, HLA-DRB1 and HLA-DRA). Genes that were elevated at 6 h, but significantly downregulated at 24 h, differed for each IFN type. This demonstrates that, in keratinocytes, a significant percentage of DEGs elicited by IFN have markedly different timing of upregulation.

## 4. Discussion

The skin serves as both a physical and immunological barrier to the entry and egress of pathogens, and both anecdotal and experimental evidence has demonstrated that skin is permissive to EBOV infection [7,8,16,20,21,22,23,24]. We show here that telomerase-immortalized NHSK-1 cells support productive EBOV infection. We similarly show that NHSK-1 cells that have stable VP30 expression support infection with EBOVΔVP30 and serve as a useful low-containment model for studying keratinocyte-virus interactions.

As a central component of the skin barrier, epidermal keratinocytes are known to possess potent innate immune defenses against viral pathogens [26,27,75]. We found that rVSV/EBOV infection of NHSK-1 cells stimulated expression of a series of ISGs. However, EBOVΔVP30 infection did not elicit endogenous antiviral responses from 6 h to 48 h, consistent with the well-established IFN-antagonistic functions of the EBOV proteins VP24 and VP35 [64,65,66,67,76]. This is also consistent with our prior observations made in EBOV-infected mice [24], where robust innate immune responses were detected in liver and adipose fat, but not in skin. Although not statistically significant, the ISG PYDC1 was most upregulated by EBOVΔVP30 infection (Figure 2 and Figure S2). PYDC1 is a negative regulator of inflammasomes [77], a pathogen recognition apparatus that elicits pyroptotic cell death and the release of pro-inflammatory cytokines. Human expression of PYDC1 is almost exclusively restricted to epithelial cells [78]. Although EBOV can stimulate inflammasome activation in Vero cells via the NLRP3 pathway to result in production of IL-1β [79], a role for the inflammasome in control of EBOV replication has not been demonstrated. Our study suggests that further exploration of cell type-specific inflammasome activation may reveal a role for this pathway in EBOV infection.

As noted above, in contrast to EBOVΔVP30, rVSV/EBOV GP elicited strong IFN responses. This is consistent with the importance of innate immune responses to rVSV/EBOV GP in its function as an effective vaccine [70,71]. In human keratinocytes, a wide range of ISGs was upregulated, including several chemokines such as CCL5 and CXCL10. IFN-β was the most upregulated gene, which would promulgate innate immune responses. In an in vivo setting, upregulation of these chemokines, along with other pro-inflammatory mediators, would likely result in recruitment of immune cells and downstream orchestration of protective adaptive immunity. Together with our previous report that rVSV/EBOV GP targets multiple cell types in human skin explants [23], these data suggest topical or subcutaneous administration of rVSV/EBOV GP as a straightforward alternative vaccine administration route to elicit protective vaccine-elicited responses against EBOV infection. In vivo exploration of EBOV-specific adaptive immunity following topical or subcutaneous administration of rVSV/EBOV GP is warranted.

Keratinocytes are responsive to type I and type II IFNs, which has largely been established in the context of autoimmune diseases [44,45,46,47]. This includes cutaneous lupus erythematosus (CLE), where keratinocytes derived from diseased skin are hyperresponsive to type I and type II IFNs [80]. However, no study has directly compared the ISGs elicited by type I, II, and III IFNs in human keratinocytes and their comparative ability to inhibit viral replication. We found that IFN-β elicited the strongest response with the greatest number of DEGs, while a subset of these was stimulated by IFN-α and IFN-λ. This overlap in gene expression is not surprising, given that ISGs stimulated by type I and type III IFNs are driven by STAT1/STAT2/IRF9 complexes [33,36]. DEGs upregulated by IFN-β and IFN-γ overlapped to a lesser extent, which is consistent with IFN-γ-elicited responses being driven by STAT1 homodimers [33].

While a large number of ISGs are elicited by IFNs, specific small subsets of ISGs may be effective against any given viral infection [81]. Notably, the EBOV inhibition observed in IFN-treated keratinocytes is relatively modest compared to IFN-mediated inhibition in other cell types. For example, prior studies from our lab have showed that 0.2 ng/mL of murine IFN-γ caused a four-fold decrease in percent infection of murine peritoneal macrophages [31]. This concentration caused no apparent inhibition in human keratinocytes in our study, where only 20 and 200 ng/mL concentrations were inhibitory and caused a modest two-fold decrease in infection (Figure 3). In another study, IFN-β caused over 50% inhibition of EBOV infection in Vero E6 cells, Huh7 cells, and human monocyte-derived macrophages at low concentrations relative to those used in our study [72]. These results support previous findings that keratinocytes have differential cell type-specific ISG expression patterns compared to macrophages that influence their antiviral capabilities [55]. Our study suggests that IFN-elicited expression programs or ISG functionality may differ in keratinocytes, leading to less effective EBOV inhibition in this cell type as compared to macrophages.

Even so, IFN-β, IFN-γ, and IFN-λ all significantly inhibit EBOV infection in keratinocytes, and our parallel transcriptomic studies allowed us to generate a focused array of candidate ISGs (Figure 4) that may contribute to IFN-mediated inhibition of EBOV in keratinocytes. This list includes several classical ISGs and pathogen-recognition receptors consistent with broad innate immune activation, such as MX1, DDX58, OAS1, and NLRC5. Several other putative antiviral ISGs were identified that had not been studied in the context of EBOV infection. Two of these identified ISGs, CMPK2 and TRIM22, modestly inhibit EBOVΔVP30 infection in NHSK-1-VP30 cells. Like other members of the TRIM family, TRIM22 is a E3 ubiquitin ligase that mediates its antiviral activity through poly-ubiquitination of viral proteins and other complex protein-protein interactions [82,83]. CMPK2 belongs to a nucleoside monophosphate kinase family that has been shown to broadly inhibit flaviviruses such as Zika virus and Dengue virus [84,85]. Our data demonstrate a potential role for these ISGs in the IFN-mediated restriction of EBOV infection in human keratinocytes. Further exploration of the antiviral mechanism of TRIM22 and CMPK2 in keratinocytes is warranted, as well as an exploration of their anti-EBOV capabilities in other critical cell types such as macrophages.

Inhibition elicited by type I and III IFNs was more robust at 24 h than at 6 h, suggesting that ISGs produced later in this 24 h period were more important for anti-EBOV IFN responses (Tables S1 and S3). In contrast, the inhibition mediated by IFN-γ was equivalent at 6 h and 24 h, suggesting that the IFN-γ-elicited ISGs driving EBOV inhibition are expressed by 6 h. Further, at 24 h, IFN-γ was less effective at blocking EBOVΔVP30 infection than IFN-β. It is possible that this is due to more limited IFN-γ receptor expression on keratinocytes; however, our data showing that virus inhibition by type I and II IFNs is equivalent at 6 h suggest that this is likely not the case (Figure 3A).

Interferons have been tested as clinical therapeutics against viral infections, with some success. Chronic hepatitis C virus (HCV) and hepatitis B virus (HBV) infections have been treated with type I IFNs for more than 30 years [86,87,88]. While IFN-γ alone was not efficacious against HBV, IFN-γ pretreatment followed by IFN-α enhanced immune responses and was associated with increased viral clearance [89]. For respiratory infections such as SARS-CoV-2, a recent meta-analysis of clinical trials concluded that type I and III IFNs were effective and were reasonably well tolerated and safe [90].

The efficacy of IFNs against Ebola virus infection has been explored in animal models. Early studies in EBOV-challenged NHPs demonstrated that IFN-α2a treatment resulted in a modest delay in viremia and time to death [91]. Separate studies found that adenovirus-vectored IFN-α alone was not protective, but it was robustly effective in both guinea pigs and NHPs when combined with a cocktail of anti-EBOV monoclonal antibodies [92]. Prophylactic delivery of adenovirus-vectored porcine IFN-α, but not IFN-λ3, one day prior to EBOV challenge strongly reduced viral loads and protected animals against disease [93]. IFN-β was also therapeutically efficacious when treatment of EBOV- or Marburg virus-infected rhesus macaques occurred within 18 hpi, significantly enhancing survival [94]. Similarly, IFN-γ treatment of mice up to 24 h after mouse-adapted EBOV challenge reduced viral loads and prevented mortality [31]. Finally, during the West African epidemic, nine EVD patients treated with IFN-β appeared to have more rapid virus clearance and faster resolution of the disease compared to an untreated retrospective cohort [30]. Combined with our new findings, we speculate that administration of topical IFN-containing creams might serve to reduce or prevent transmission of EBOV from the skin’s surface. However, caution should be exercised in the application of findings to humans, based on a prior report demonstrating that elevated levels of IFN-α and IFN-γ in EBOV-infected humans correlated with worse outcomes [95]. Overall, these findings suggest that the therapeutic value of IFNs, particularly in combination with other antiviral therapies, should be explored further.

## Data Availability

Most of the data are contained within the article or Supplementary Material. Raw sequencing data including FASTQ files and count matrices are available on NCBI Gene Expression Omnibus (GEO) with the accession number GSE309699. Further inquiries can be directed to the corresponding author.

## Supplementary Materials

The following supporting information can be downloaded at: https://www.mdpi.com/article/10.3390/v17121577/s1, Figure S1: EBOVΔVP30 Infection Gating Strategy; Figure S2: rVSV/EBOV elicits antiviral responses in human keratinocytes; Figure S3: EBOVΔVP30 does not elicit antiviral responses in human keratinocytes; Figure S4: Timing of antiviral responses elicited by rVSV/EBOV and EBOVΔVP30; Figure S5: Treatment with IFN-β, IFN-γ, and IFN-λ inhibits rVSV/EBOV replication in human keratinocytes and EBOVΔVP30 in multiple NHSK-1-VP30 clones; Figure S6: Interferons elicit comparable ISG expression in NHSK-1 and NHSK-1-VP30 cells; Figure S7: Interferons elicit a range of overlapping and unique pathways in human keratinocytes; Table S1: IFN-β-stimulated DEGs unique to 6- or 24-h treatment; Table S2: IFN-γ-stimulated DEGs unique to 6- or 24-hour treatment; Table S3: IFN-λ-stimulated DEGs unique to 6- or 24-h treatment.

## Abbreviations

The following abbreviations are used in this manuscript:

EBOV	*Orthoebolavirus zairense* or Zaire Ebola virus
EBOVΔVP30	EBOV lacking expression of VP30
rVSV/EBOV GP	Recombinant vesicular stomatitis virus encoding EBOV GP
IFN	Interferon
NHSK-1	Normal human skin keratinocyte 1
ISG	Interferon-stimulated gene
DRC	Democratic Republic of the Congo
NHP	Non-human primate
WHO	World Health Organization
EVD	Ebola virus disease
HPV	Human papillomavirus
IFNAR	Interferon-α/β receptor
STAT	Signal transducer and activator of transcription
CMPK	Cytidylate monophosphate kinase 2
TRIM	Tripartite motif protein
IRF	Interferon regulatory factor
DC	Dendritic cell
GFP	Green fluorescent protein
MOI	Multiplicity of infection
RT-qPCR	Real-time quantitative polymerase chain reaction
DEG	Differentially expressed gene
PCA	Principal component analysis
CLE	Cutaneous lupus erythematosus
MAP	Mitogen-activated protein kinase
HSV-1	Herpes simplex virus 1
HCV	Hepatitis C virus
HBV	Hepatitis B virus
