# Supplementary material for: Interferons Inhibit Ebola Virus Infection of Human Keratinocytes
Source: Viruses. 2025 Dec 2;17(12):1577. doi: 10.3390/v17121577 (PMC12737376; doi:10.3390/v17121577)
Supplement: Supplementary file 1 [file viruses-17-01577-s001.zip › viruses-3926161-supplementary.pdf]

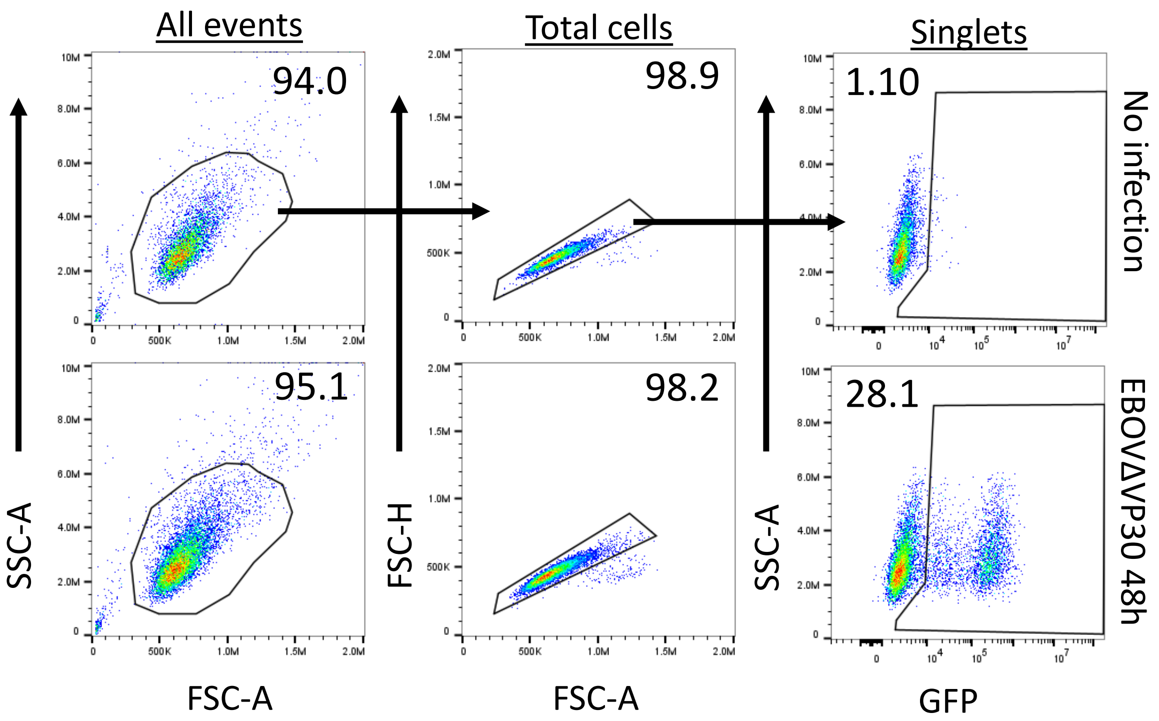
All events
Total cells
Singlets
94.0
98.9
1.10
95.1
98.2
28.1
No infection
EBOVΔVP30 48h
SSC-A
FSC-H
SSC-A
FSC-A
FSC-A
GFP

Supplemental Figure 1: EBOVΔVP30 Infection Gating Strategy

Related to Figure 1E and Figure 3.

NHSK-1 cells were infected with increasing concentrations of EBOVΔVP30-GFP (MOI=0.1, 1, and 10). Infection was evaluated via the percentage of single keratinocytes expressing GFP. Representative gating strategy is shown for uninfected cells (top row) and for cells infected with MOI=1 EBOVΔVP30 (bottom row).

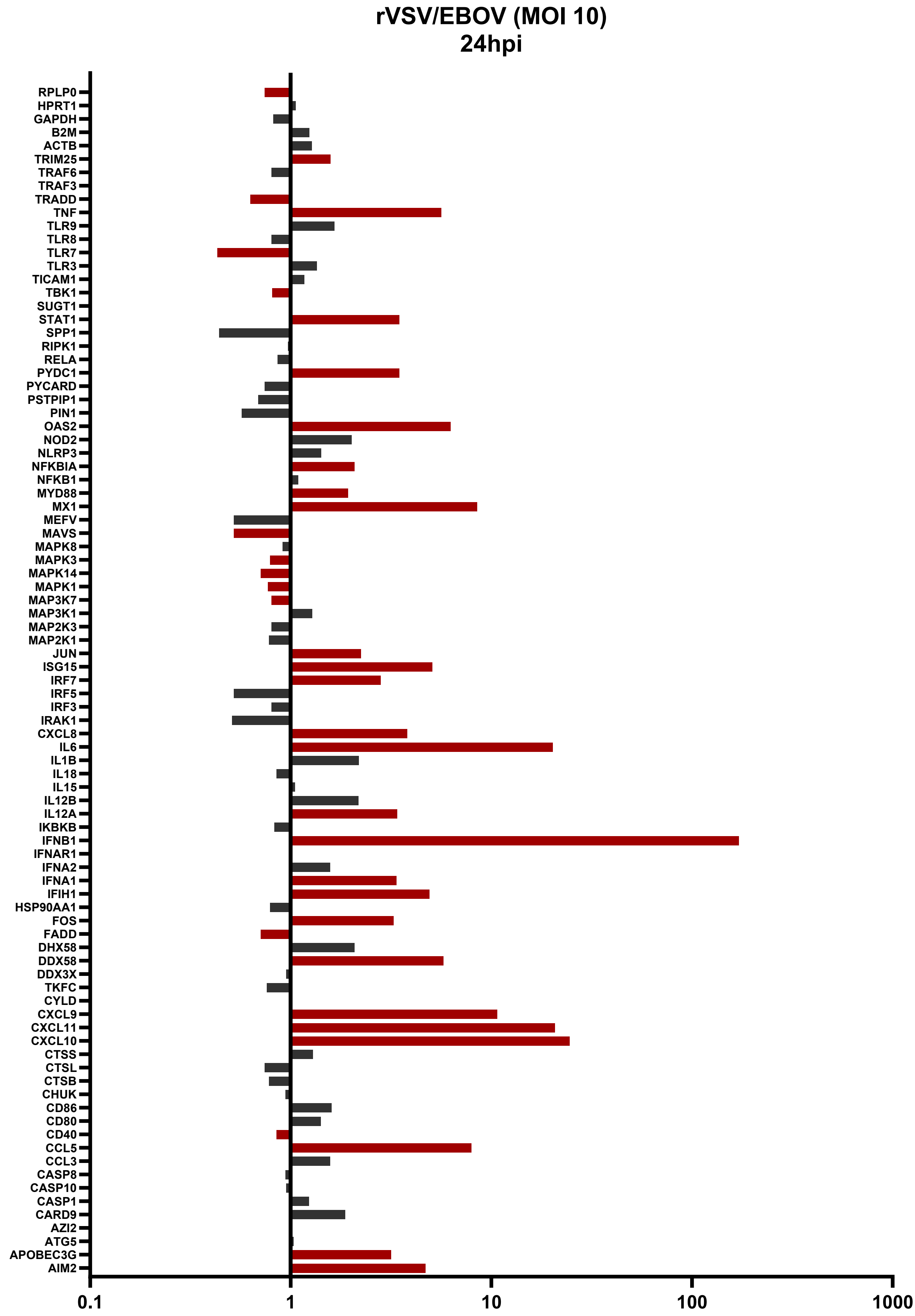

rVSV/EBOV (MOI 10)
24hpi
RPLP0
HPRT1
GAPDH
B2M
ACTB
TRIM25
TRAF6
TRAF3
TRADD
TNF
TLR9
TLR8
TLR7
TLR3
TICAM1
TBK1
SUGT1
STAT1
SPP1
RIPK1
RELA
PYDC1
PYCARD
PSTPIP1
PIN1
OAS2
NOD2
NLRP3
NFKBIA
NFKB1
MYD88
MX1
MEFV
MAVS
MAPK8
MAPK3
MAPK14
MAPK1
MAP3K7
MAP3K1
MAP2K3
MAP2K1
JUN
ISG15
IRF7
IRF5
IRF3
IRAK1
CXCL8
IL6
IL1B
IL18
IL15
IL12B
IL12A
IKBKB
IFNB1
IFNAR1
IFNA2
IFNA1
IFIH1
HSP90AA1
FOS
FADD
DHX58
DDX58
DDX3X
TKFC
CYLD
CXCL9
CXCL11
CXCL10
CTSS
CTSL
CTSB
CHUK
CD86
CD80
CD40
CCL5
CCL3
CASP8
CASP10
CASP1
CARD9
AZI2
ATG5
APOBEC3G
AIM2
0.1
1
10
100
1000

Supplemental Figure 2: rVSV/EBOV GP elicits antiviral responses in human keratinocytes.

Related to Figure 2.

NHSK-1 cells expressing EBOV VP30 were infected with MOI=10 rVSV/EBOV or left uninfected. RNA was isolated at 24 hpi for assessment of antiviral gene expression via PCR.

Shown are the fold changes in infected cells over uninfected baseline expression in 84 genes associated with human antiviral responses. Red bars denote significance as defined by $p<0.05$ via Student's t-test. Data representative of 2 independent experiments, each with 3 independent biological replicates.

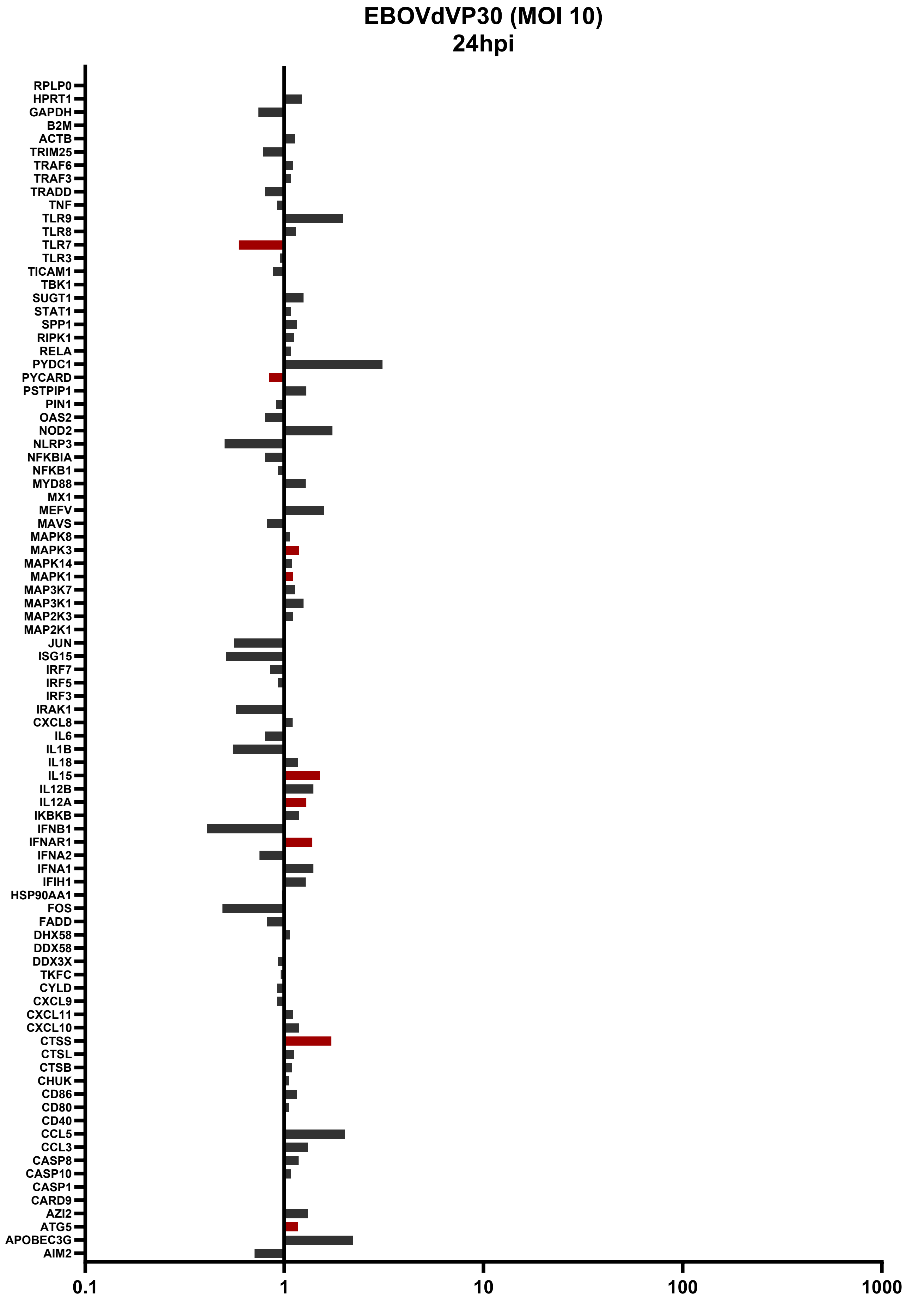
EBOVdVP30 (MOI 10)
24hpi
RPLP0
HPRT1
GAPDH
B2M
ACTB
TRIM25
TRAF6
TRAF3
TRADD
TNF
TLR9
TLR8
TLR7
TLR3
TICAM1
TBK1
SUGT1
STAT1
SPP1
RIPK1
RELA
PYDC1
PYCARD
PSTPIP1
PIN1
OAS2
NOD2
NLRP3
NFKBIA
NFKB1
MYD88
MX1
MEFV
MAVS
MAPK8
MAPK3
MAPK14
MAPK1
MAP3K7
MAP3K1
MAP2K3
MAP2K1
JUN
ISG15
IRF7
IRF5
IRF3
IRAK1
CXCL8
IL6
IL1B
IL18
IL15
IL12B
IL12A
IKBKB
IFNB1
IFNAR1
IFNA2
IFNA1
IFIH1
HSP90AA1
FOS
FADD
DHX58
DDX58
DDX3X
TKFC
CYLD
CXCL9
CXCL11
CXCL10
CTSS
CTSL
CTSB
CHUK
CD86
CD80
CD40
CCL5
CCL3
CASP8
CASP10
CASP1
CARD9
AZI2
ATG5
APOBEC3G
AIM2
0.1
1
10
100
1000

Supplemental Figure 3: EBOVΔVP30 does not elicit antiviral responses in human keratinocytes.

Related to Figure 2.

NHSK-1 cells expressing EBOV VP30 were infected with MOI=10 EBOVΔVP30 or left uninfected. RNA was isolated at 24 hpi for assessment of antiviral gene expression via PCR.

Shown are the fold changes in infected cells over uninfected baseline expression in 84 genes associated with human antiviral responses. Red bars denote significance as defined by $p<0.05$ via Student's t-test. Data representative of 2 independent experiments, each with 3 independent biological replicates.

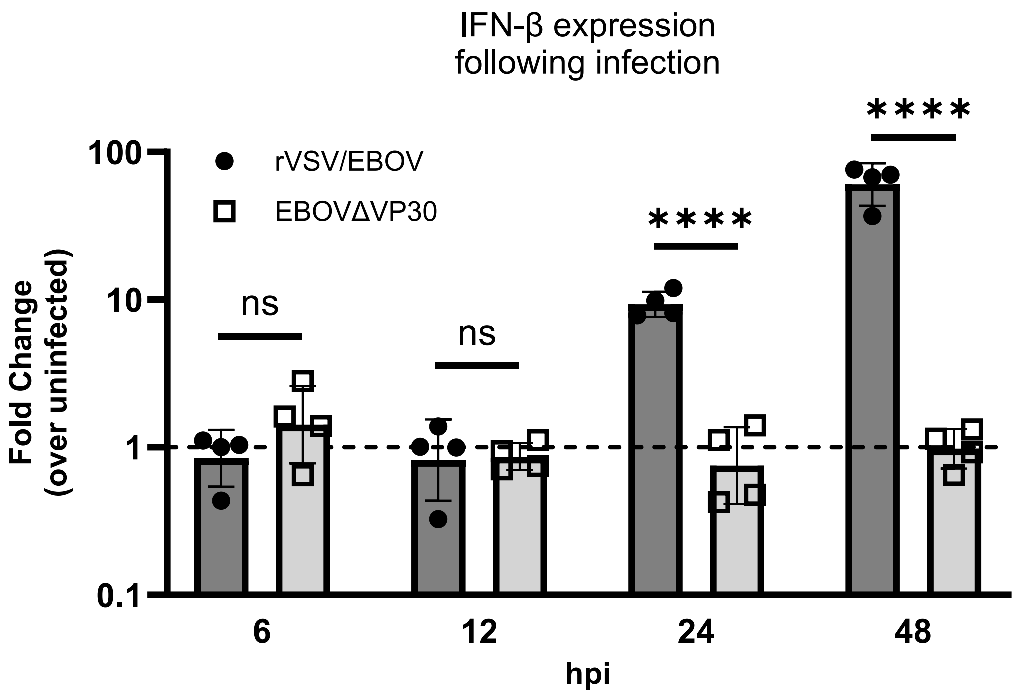
IFN-β expression
following infection
rVSV/EBOV
EBOVΔVP30
Fold Change
(over uninfected)
100
10
1
0.1
ns
ns
****
****
6
12
24
48
hpi

Supplemental Figure 4: Timing of antiviral responses elicited by rVSV/EBOV and EBOVΔVP30.

Related to Figure 2.

NHSK-1 cells expressing EBOV VP30 were infected with rVSV/EBOV or EBOVΔVP30 (MOI = 10) or left uninfected. At 6-, 12-, 24-, and 48-hpi, RNA was isolated and assessed by RT-qPCR for expression of *Ifnb*. Expression values were normalized to *Gapdh* expression. (n=4, data obtained in one experiment)

Data was analyzed for significance using a Student's t-test and is represented as mean fold change over uninfected ± SD. *p<0.05, **p<0.01, ***p<0.001, ****p<0.0001, ns = not significant.

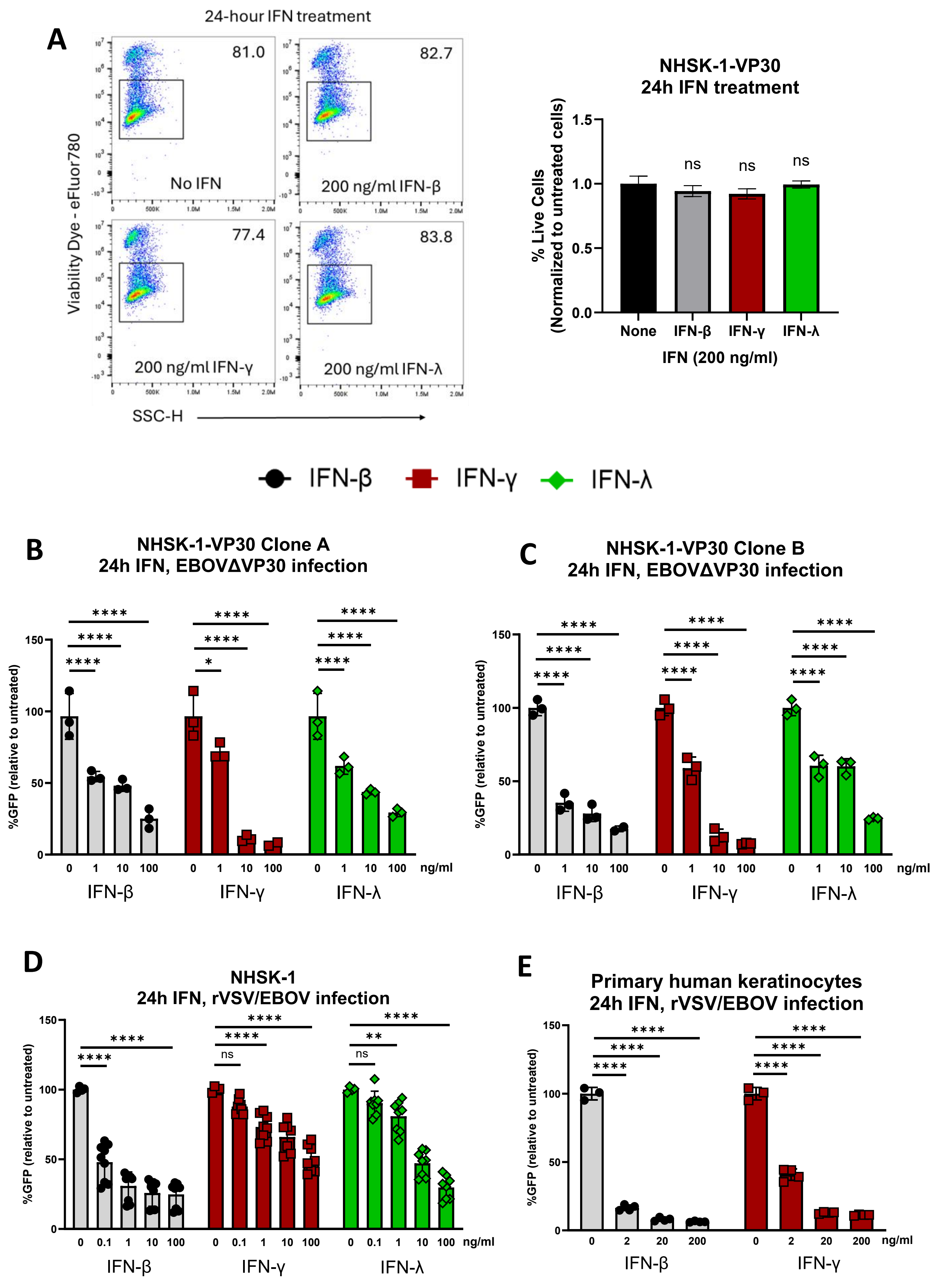
A
24-hour IFN treatment
81.0
82.7
77.4
83.8
No IFN
200 ng/ml IFN-β
200 ng/ml IFN-γ
200 ng/ml IFN-λ
Viability Dye - eFluor780
SSC-H
NHSK-1-VP30
24h IFN treatment
% Live Cells
(Normalized to untreated cells)
ns
None
IFN-β
IFN-γ
IFN-λ
IFN (200 ng/ml)
IFN-β
IFN-γ
IFN-λ
B
NHSK-1-VP30 Clone A
24h IFN, EBOVΔVP30 infection
%GFP (relative to untreated)
ng/ml
C
NHSK-1-VP30 Clone B
24h IFN, EBOVΔVP30 infection
D
NHSK-1
24h IFN, rVSV/EBOV infection
E
Primary human keratinocytes
24h IFN, rVSV/EBOV infection

Supplemental Figure 5: Treatment with IFN-β, IFN-γ, and IFN-λ inhibits rVSV/EBOV replication in human keratinocytes and EBOVΔVP30 in multiple NHSK-1-VP30 clones

Related to Figure 3.

(A) NHSK-1-VP30 cells were treated with 200 ng/ml of IFN-β, IFN-γ, or IFN-λ, and cell viability was assessed by flow at 24 hours of treatment. Representative plots are shown (left), and percentage of live cells is indicated in the top right corner. Summarized live cell percentages were normalized to untreated cells and are shown (right). (n=4, data obtained in one experiment).

(B-C) NHSK-1-VP30 clones A and B were treated with increasing concentrations of IFN-β, IFN-γ, or IFN-λ for 24 hours, then infected with MOI=10 EBOVΔVP30. Infection was evaluated at 48 hpi by flow cytometry. (n=3, data obtained in one experiment).

(D) NHSK-1 cells were treated with increasing concentrations of IFN-β, IFN-γ, or IFN-λ for 24 hours, then infected with MOI=10 rVSV/EBOV GP. Infection was evaluated at 24-hpi by flow cytometry (left) and AUC is shown (right). (n=8-9, data pooled from 3 independent experiments).

(E) Primary human keratinocytes were treated with increasing concentrations of IFN-β or IFN-γ for 24 hours, then infected with MOI=10 rVSV/EBOV GP. Infection was evaluated at 24-hpi by flow cytometry. (n=4, data obtained in one experiment).

Live cell percentages, relative %GFP, and calculated AUC values were analyzed using a one-way ANOVA. AUC values are represented as mean ± SEM. Relative %GFP were analyzed using a two-way ANOVA are represented as mean ± SD. *$p<0.05$, **$p<0.01$, ***$p<0.001$, ****$p<0.0001$, ns = not significant.

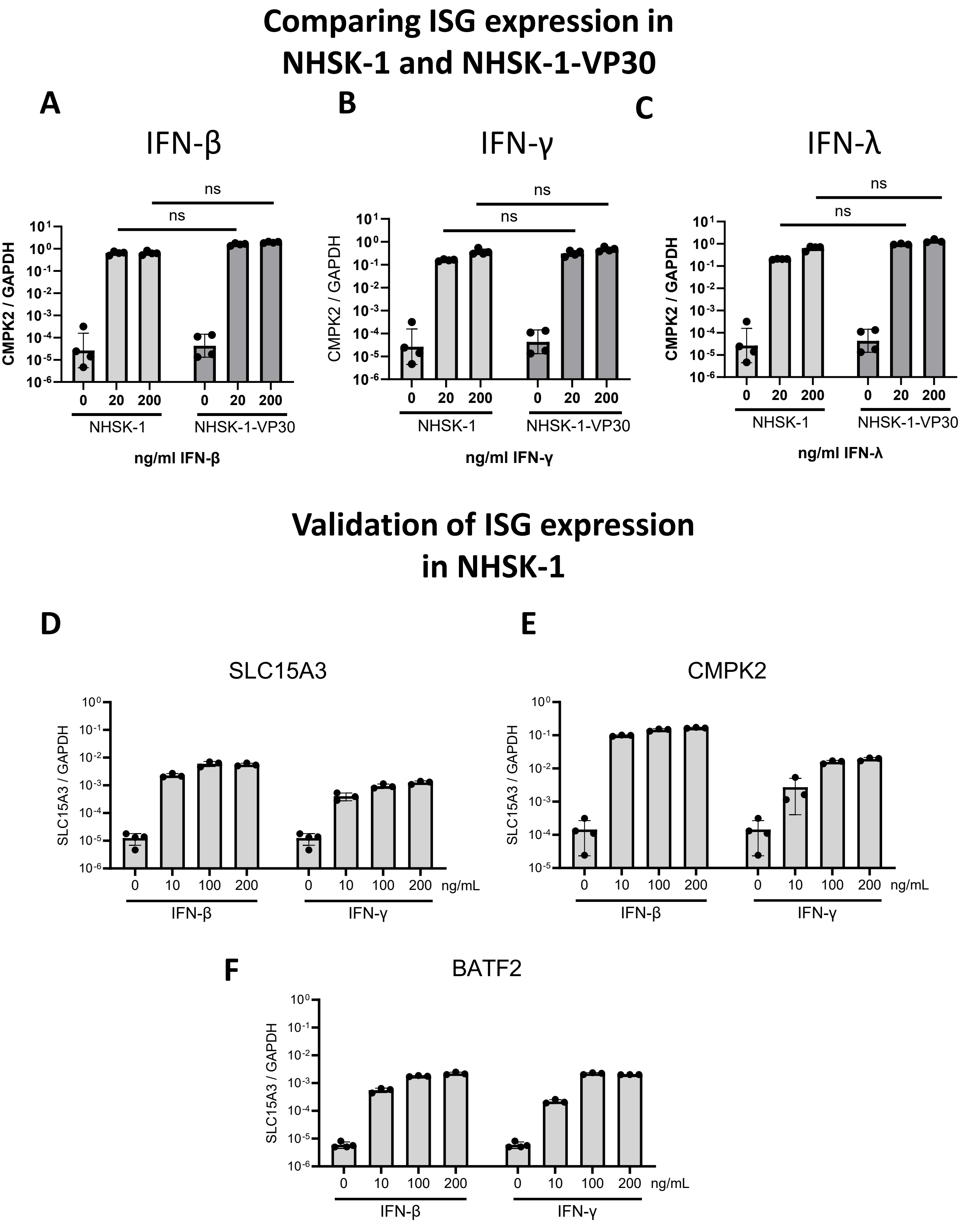
Comparing ISG expression in NHSK-1 and NHSK-1-VP30
A
IFN-β
CMPK2 / GAPDH
ns
ns
0 20 200 0 20 200
NHSK-1
NHSK-1-VP30
ng/ml IFN-β
B
IFN-γ
CMPK2 / GAPDH
ns
ns
0 20 200 0 20 200
NHSK-1
NHSK-1-VP30
ng/ml IFN-γ
C
IFN-λ
CMPK2 / GAPDH
ns
ns
0 20 200 0 20 200
NHSK-1
NHSK-1-VP30
ng/ml IFN-λ
Validation of ISG expression in NHSK-1
D
SLC15A3
SLC15A3 / GAPDH
0 10 100 200 0 10 100 200 ng/mL
IFN-β
IFN-γ
E
CMPK2
SLC15A3 / GAPDH
0 10 100 200 0 10 100 200 ng/mL
IFN-β
IFN-γ
F
BATF2
SLC15A3 / GAPDH
0 10 100 200 0 10 100 200 ng/mL
IFN-β
IFN-γ

Supplemental Figure 6: Interferons elicit comparable ISG expression in NHSK-1 and NHSK-1-VP30 cells.

Related to Figure 4.

(A-C) RT-qPCR amplification of CMPK2 mRNA in either NHSK-1 cells (light gray bars) or NHSK-1-VP30 cells (dark grey bars) treated for 24 hours with IFN-β **(A)**, IFN-γ **(B)**, or IFN-λ **(C)**. Expression was normalized to *GAPDH.* (n=4, data obtained in one experiment)

(D-F) RT-qPCR amplification of *SLC154A3* **(D)*,*** *CMPK2* **(E),** and *BATF2* **(F)** mRNA in NHSK-1 cells treated for 24 hours IFN-β or IFN-γ. Expression was normalized to *GAPDH.* (n=5, data obtained in one experiment)

Normalized expression values were analyzed using a two-way ANOVA and are represented as mean ± SD. *p<0.05, **p<0.01, ***p<0.001, ****p<0.0001, ns = not significant.

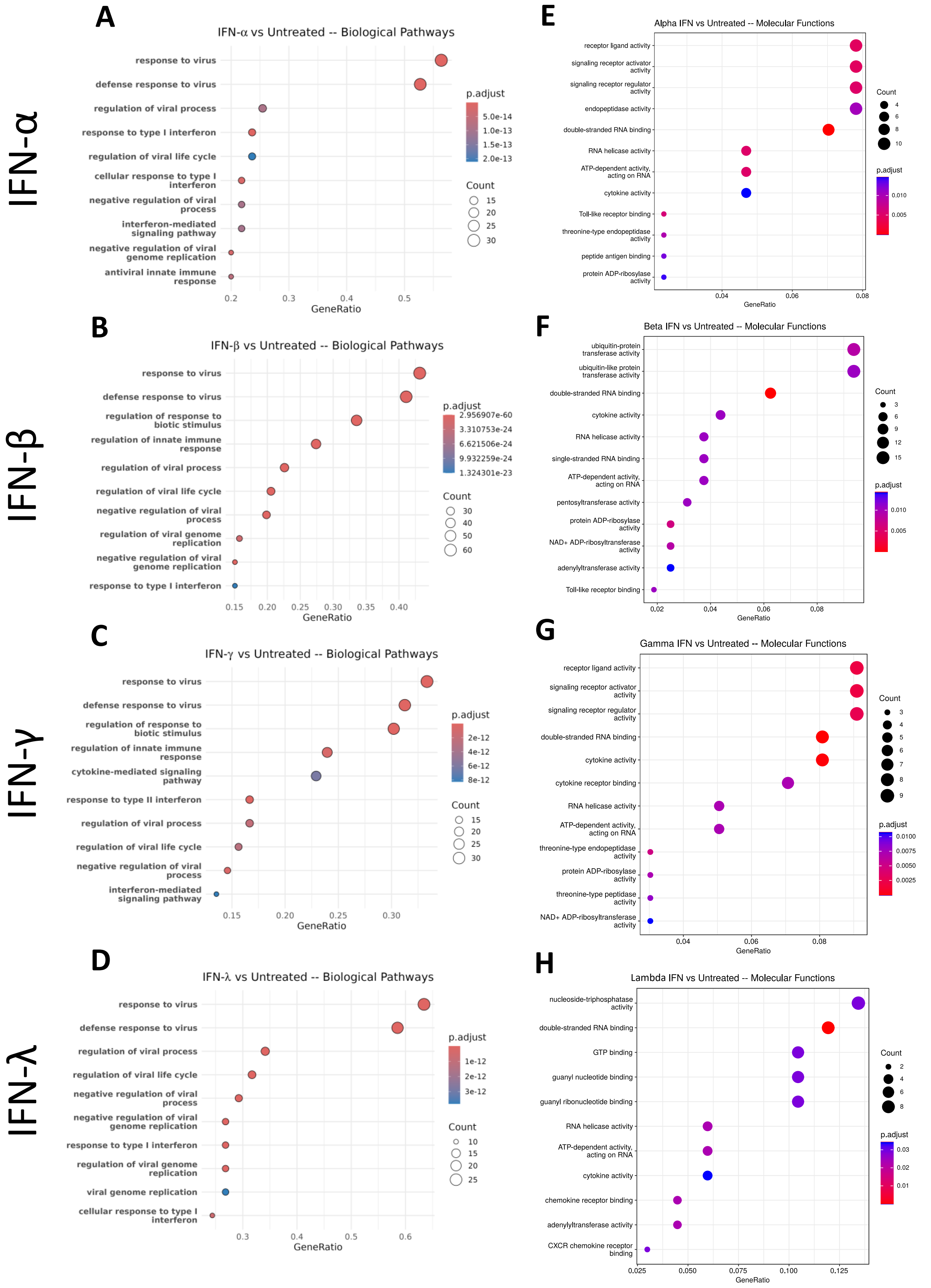
A
IFN-α
IFN-α vs Untreated -- Biological Pathways
response to virus
defense response to virus
regulation of viral process
response to type I interferon
regulation of viral life cycle
cellular response to type I interferon
negative regulation of viral process
interferon-mediated signaling pathway
negative regulation of viral genome replication
antiviral innate immune response
GeneRatio
p.adjust
Count
B
IFN-β
IFN-β vs Untreated -- Biological Pathways
response to virus
defense response to virus
regulation of response to biotic stimulus
regulation of innate immune response
regulation of viral process
regulation of viral life cycle
negative regulation of viral process
regulation of viral genome replication
negative regulation of viral genome replication
response to type I interferon
GeneRatio
p.adjust
Count
C
IFN-γ
IFN-γ vs Untreated -- Biological Pathways
response to virus
defense response to virus
regulation of response to biotic stimulus
regulation of innate immune response
cytokine-mediated signaling pathway
response to type II interferon
regulation of viral process
regulation of viral life cycle
negative regulation of viral process
interferon-mediated signaling pathway
GeneRatio
p.adjust
Count
D
IFN-λ
IFN-λ vs Untreated -- Biological Pathways
response to virus
defense response to virus
regulation of viral process
regulation of viral life cycle
negative regulation of viral process
negative regulation of viral genome replication
response to type I interferon
regulation of viral genome replication
viral genome replication
cellular response to type I interferon
GeneRatio
p.adjust
Count
E
Alpha IFN vs Untreated -- Molecular Functions
receptor ligand activity
signaling receptor activator activity
signaling receptor regulator activity
endopeptidase activity
double-stranded RNA binding
RNA helicase activity
ATP-dependent activity, acting on RNA
cytokine activity
Toll-like receptor binding
threonine-type endopeptidase activity
peptide antigen binding
protein ADP-ribosylase activity
GeneRatio
Count
p.adjust
F
Beta IFN vs Untreated -- Molecular Functions
ubiquitin-protein transferase activity
ubiquitin-like protein transferase activity
double-stranded RNA binding
cytokine activity
RNA helicase activity
single-stranded RNA binding
ATP-dependent activity, acting on RNA
pentosyltransferase activity
protein ADP-ribosylase activity
NAD+ ADP-ribosyltransferase activity
adenylyltransferase activity
Toll-like receptor binding
GeneRatio
Count
p.adjust
G
Gamma IFN vs Untreated -- Molecular Functions
receptor ligand activity
signaling receptor activator activity
signaling receptor regulator activity
double-stranded RNA binding
cytokine activity
cytokine receptor binding
RNA helicase activity
ATP-dependent activity, acting on RNA
threonine-type endopeptidase activity
protein ADP-ribosylase activity
threonine-type peptidase activity
NAD+ ADP-ribosyltransferase activity
GeneRatio
Count
p.adjust
H
Lambda IFN vs Untreated -- Molecular Functions
nucleoside-triphosphatase activity
double-stranded RNA binding
GTP binding
guanyl nucleotide binding
guanyl ribonucleotide binding
RNA helicase activity
ATP-dependent activity, acting on RNA
cytokine activity
chemokine receptor binding
adenylyltransferase activity
CXCR chemokine receptor binding
GeneRatio
Count
p.adjust

Supplemental Figure 7: Interferons elicit a range of overlapping and unique pathways in human keratinocytes

NHSK-1 cells were treated 100 ng/ml of IFN-β or IFN-γ, 18.8 ng/ml of IFN-λ, or 2.47 ng/ml of IFN-α. RNA was isolated for sequencing at 6 or 24 hours of IFN treatment. Sequencing was performed twice with four technical replicates for each IFN treatment. Biological pathway (left) and molecular function (right) GO Pathway analysis results for IFN-α (A, E), IFN-β (B,F), IFN-γ (C,G), and IFN-λ (D,H).

**Table S1:** IFN-β-stimulated DEGs unique to 6- or 24-hour treatment		
**6-hour treatment**	**24-hour treatment**	
APOBEC3F	ACKR4	MDK
CASP7	ACO1	MOV10
CAVIN2	AIM2	NEBL
CD274	APOBEC3B	NFASC
CNP	B2M	NID1
FLT3LG	BISPR	ODF3B
GCH1	BTN3A2	PARP3
HAPLN3	CIB2	PLAAT2
HCAR2	CLCA3P	PLAAT4
IL22RA1	CLTRN	PPM1K
IRF2	CTSO	PSMB10
IRF9	CX3CL1	RET
OGFR	CXCL9	RETREG1
PDZD2	DANCR	RRAD
POU3F1	DHRS2	RTP4
TGFB2	DNER	SAA1
THSD1	EXOC3L1	SEMA3A
TMEM171	GSDMD	SEMA3D
TRIM25	HCAR3	SIRPB2
XRN1	HCP5	ST3GAL5
ZBTB42	HES4	SUSD4
ZC3HAV1	HEXD	TAPBPL
	HLA-A	TCN2
	HLA-C	TIMP2
	IFI30	TMEM229B
	IFITM2	TNFSF15
	IFITM3	VSIG10L
	IL18R1	
	KRT75	
	LINC02574	
	LY6E	
	MAP2	

Table S1: IFN-β-stimulated DEGs unique to 6- or 24-hour treatment

NHSK-1 cells were treated with 100 ng/ml of IFN-β. RNA was isolated for sequencing at 6 or 24 hours of IFN treatment. Sequencing was performed twice with three technical replicates.

Table shows DEGs uniquely expressed at either 6 hours or 24 hours of IFN treatment. Only genes meeting the highly stringent criteria of $\log_2$fold change > 2 and an adjusted p-value ≤ 10^{-32} are shown.

Related to Figure 5A.

Table S2: IFN-γ-stimulated DEGs unique to 6- or 24-hour treatment

**6-hour treatment**	**24-hour treatment**	
ALPK1	LAP3	CXCL10
JAK2	CX3CL1	SAA1
HAS3	TYMP	RNF213
ZC3HAV1	PARP3	HLA-DQB1
DKK1	CEACAM1	CIITA
INHBA	FYB1	MX2
SOX9	FLT3LG	MUC1
STEAP4	PSME1	GSAP
HELZ2	PSME2	BTN3A2
IL33	GSDMD	ISG15
FAM117B	LGALS3BP	HLA-DRB1
IL22RA1	IL1R2	HLA-DMA
IFIT5	IL18R1	TAP2
ADAMTS1	FBXO6	HLA-DRA
IFI16	ACKR4	CARD16
IL15	ACE2	HLA-C
CASP7	BST2	HLA-E
MLKL	ASS1	HLA-F
SOCS3	PLAAT4	HCP5
CA13	IL18BP	GBP1P1
ZFP57	IFI44L	HLA-DPA1
DUBR	IFI44	HLA-B
	TAPBPL	CFB
	SLC38A4	HCAR3
	IFITM3	CTSO
	GBP5	
	TNFRSF14	
	CXCR1	
	AIM2	
	ERAP1	
	ERAP2	
	IFI27	
	RRAD	
	B2M	

Table S2: IFN-γ-stimulated DEGs unique to 6- or 24-hour treatment

NHSK-1 cells were treated with 100 ng/ml of IFN-γ. RNA was isolated for sequencing at 6 or 24 hours of IFN treatment. Sequencing was performed twice with three technical replicates.

Table shows DEGs uniquely expressed at either 6 hours or 24 hours of IFN treatment. Only genes meeting the highly stringent criteria of log_2fold change > 2 and an adjusted p-value ≤ 10^{-32} are shown.

Related to Figure 5B.

**Table S3:** IFN-λ-stimulated DEGs unique to 6- or 24-hour treatment	
**6-hour treatment**	**24-hour treatment**
OAS3	CD68
STAT1	ACE2
TRIM5	PLAAT4
SP110	SERPING1
DDX60	CTSS
IFI44	IFI27
PARP14	RNF213
EXOC3L1	IFITM2
IRF7	HLA-F
SAMD9	HCP5
APOL6	HLA-B
	APOBEC3G
	PSMB9
	CFB
	CFH

Table S3: IFN-λ-stimulated DEGs unique to 6- or 24-hour treatment

NHSK-1 cells were treated with 18.8 ng/ml of IFN-λ. RNA was isolated for sequencing at 6 or 24 hours of IFN treatment. Sequencing was performed twice with three technical replicates.

Table shows DEGs uniquely expressed at either 6 hours or 24 hours of IFN treatment. Only genes meeting the highly stringent criteria of $\log_2$fold change > 2 and an adjusted p-value ≤ 10^{-32} are shown.

Related to Figure 5C.
